# Effects of Combined Blood Flow Restriction and Neuromuscular Electrical Stimulation Versus Blood Flow Restriction Alone: A Systematic Review and Meta-Analysis

**DOI:** 10.3390/jcm15218725

**Published:** 2026-11-08

**Authors:** Shams Al-dawoodi, Ebrahim Safaii, Mohamedanas Mohamedfaruk Patni, Biji Thomas George, Subhranshu Sekhar Kar, Rajani Dube, Ramya Kundayi Ravi, Rikhil Rajiv

**Affiliations:** 1 RAK College of Medical Sciences, Ras Al Khaimah Medical and Health Sciences University (RAKMHSU), Ras Al Khaimah 11172, United Arab Emirates; shams.24901116@rakmhsu.ac.ae (S.A.-d.); rikhil.24901098@rakmhsu.ac.ae (R.R.); 2 Department of Community Medicine, RAK College of Medical Sciences, RAK Medical and Health Sciences University, Ras Al Khaimah 11172, United Arab Emirates; 3 Department of Surgery, RAK College of Medical Sciences, Ras Al Khaimah Medical and Health Sciences University, Ras Al Khaimah 11172, United Arab Emirates; biji@rakmhsu.ac.ae; 4 Department of Paediatrics, RAK College of Medical Sciences, Ras Al Khaimah Medical and Health Sciences University, Ras Al Khaimah 11172, United Arab Emirates; subhranshu.kar@rakmhsu.ac.ae; 5 Department of Obstetrics and Gynaecology, RAK College of Medical Sciences, Ras Al Khaimah Medical and Health Sciences University, Ras Al Khaimah 11172, United Arab Emirates; rajani.dube@rakmhsu.ac.ae; 6 RAK College of Nursing, Ras Al Khaimah Medical and Health Sciences University, Ras Al Khaimah 11172, United Arab Emirates; ramya@rakmhsu.ac.ae

**Keywords:** blood flow restriction, neuromuscular electrical stimulation, muscle strength, hypertrophy, systematic review, meta-analysis

## Abstract

**Background/Objectives:** Blood flow restriction (BFR) training is increasingly combined with neuromuscular electrical stimulation (NMES) in musculoskeletal rehabilitation to augment strength and hypertrophy, but whether NMES adds benefit beyond BFR alone remains uncertain. This systematic review and meta-analysis compared combined BFR + NMES with BFR alone for muscle strength, muscle morphology (mass, thickness, cross-sectional area), neuromuscular activation, and functional performance. **Methods:** Randomized trials comparing simultaneous BFR + NMES with BFR alone were eligible. MEDLINE via PubMed, Scopus, the Cochrane Central Register of Controlled Trials, and Google Scholar were searched from inception through 15 September 2026. Two reviewers independently screened studies and extracted data; risk of bias was assessed with Cochrane RoB 2 and certainty with GRADE. Random-effects meta-analysis was restricted to independent parallel-group comparisons; crossover and limb-randomized results were pooled only when paired effect estimates and variances were available, otherwise synthesized narratively. The protocol was registered in PROSPERO (CRD420251076162). **Results:** Five randomized trials involving 174 participants were included. The studies represented healthy-participant training, experimental limb disuse, acute physiological responses, and low-load training in football players with knee osteoarthritis. Quantitative synthesis of torque-based strength found no clear difference between BFR + NMES and BFR alone (MD −41.78 Nm, 95% CI −396.68 to 313.12; *p* = 0.375; 2 trials, 52 participants), with substantial statistical heterogeneity. Cross-sectional area and rectus femoris EMG RMS were each reported by only two trials and were synthesized narratively. Certainty of evidence was very low across all outcomes. **Conclusions:** Current evidence does not establish a consistent additional benefit of adding NMES to BFR. The pooled torque-based estimate is exploratory because of the small evidence base, substantial statistical heterogeneity, imprecision, and differences in clinical context and outcome measurement. A possible effect on preserving muscle mass during short-term experimental disuse remains preliminary. None of the included trials directly investigated BFR + NMES following anterior cruciate ligament reconstruction (ACLR), and its effectiveness in this population remains undetermined.

## 1. Introduction

Musculoskeletal injury or immobilization causes rapid muscle atrophy and loss of strength with significant functional consequences for mobility and quality of life [1]. For instance, knee surgery is related to significant knee-extensor strength deficits in the early postoperative period, and short-term knee immobilization in healthy volunteers (5–14 days) leads to measurable reductions in quadriceps cross-sectional area (CSA) and one-repetition maximum (1RM) strength; these disuse-induced losses of muscle mass and function are difficult to reverse and often persist despite standard rehabilitation efforts [1,2]. Thus, strategies that reduce atrophy and maintain muscle strength during immobilization are essential components of musculoskeletal rehabilitation [1].

Blood flow restriction (BFR) training is a technique that uses a pneumatic cuff or elastic wrap on a limb to reduce arterial inflow while partially occluding venous outflow, creating local hypoxia, blood pooling, and significant metabolic stress on the working musculature [3]. This metabolically stressful environment (hypoxia, metabolite accumulation, and increased intramuscular pressure) can be induced even at low external loads and constitutes a central part of the physiological rationale for BFR [4,5]. Meta-analytic and conceptual work [4,5] supports the proposition that hypertrophic and strength adaptations can occur at low mechanical loads when a critical level of metabolic stress is achieved. Mechanistic studies have shown that low-load BFR exercise increases phosphorylation of S6 kinase 1 (S6K1) and activation of mammalian target of rapamycin complex 1 (mTORC1) and results in increased muscle protein synthesis, despite low mechanical tension, similar to that seen with traditional heavy-load resistance training [6,7].

In clinical practice, low-load BFR protocols (typically approximately 20–40% 1RM) cause large increases in muscle hypertrophy and strength. Results on hypertrophy are often comparable to high loads (≥65% 1RM), but maximal strength adaptations are still superior for heavy-load exercise [8,9,10]. More importantly, low-load BFR produces superior adaptations more consistently than comparable low-load resistance training without occlusion, which has led to increased use of this intervention in the rehabilitation setting, in which joint loading must be minimized [8,9]. Safety data suggest that BFR is well tolerated with appropriate use, with minor adverse events (e.g., subcutaneous hemorrhage or bruising) more common than major complications and serious events (e.g., venous thromboembolism or rhabdomyolysis) being rare when supervised [3,11]. However, meticulous pre-participation screening and individualized pressure prescription are crucial, and the inconsistency in cuff width, pressure prescription, and training protocols continues to be an important limitation in the literature [3].

Neuromuscular electrical stimulation (NMES) consists of the application of externally generated currents to elicit involuntary muscle contractions by depolarizing peripheral motor axons (or the muscle membrane itself if the innervation is damaged), thereby reducing reliance on voluntary corticospinal drive [12]. NMES recruits motor units through externally applied electrical currents, producing a recruitment pattern that differs from voluntary activation and is often spatially fixed and relatively synchronous. Unlike the orderly recruitment typically associated with voluntary contractions, electrically evoked recruitment is largely nonselective and depends on factors such as electrode placement and stimulation parameters [13,14]. NMES has been found to decrease disuse-related atrophy and maintain muscle-fiber CSA during immobilization or unloading in experimental and clinical studies [15]. Because NMES generates contractions that are supramaximal to a patient’s voluntary ability, it is particularly helpful when voluntary activation is restricted by pain, joint effusion, or arthrogenic muscle inhibition [16]. In order to enhance muscle strength recovery, NMES has been extensively utilized in orthopedic and neurologic rehabilitation, such as following total knee arthroplasty (TKA) and stroke rehabilitation [15,16,17]. NMES, when used in conjunction with exercise, reduces early postoperative quadriceps weakness following knee surgery and can result in moderate strength improvements when compared to exercise alone, according to systematic reviews and meta-analyses; hypertrophic effects are variable and frequently less than those of active resistance training [17]. NMES has practical constraints as well. The high current intensities needed to produce powerful contractions may cause discomfort or skin irritation that restricts tolerance and adherence, and its synchronous recruitment pattern causes quick exhaustion, limiting the length of the session [13,14,18]. Moreover, transfer to complex voluntary movements may be limited because NMES contractions are frequently non-functional (open-chain) [13,15,19].

The combination of BFR and NMES (BFR + NMES) has been suggested to cause muscular contractions in situations when voluntary activation is impaired (BFR + NMES) [3,20]. The two mechanisms are believed to be complementary: NMES directly recruits motor units regardless of central drive, whereas BFR causes local hypoxia and metabolite buildup, promoting intracellular anabolic pathways [19,21]. It has been proposed that NMES may partially offset the loss of voluntary activation during BFR by directly engaging motor units and delivering additive or synergistic neuromuscular and hypertrophic stimuli. Nevertheless, there is conflicting evidence of BFR-induced deficits in neuronal conduction or muscle activation, and it appears to rely on cuff pressure, occlusion time, and methodological variables. As a result, any synergistic connection should be considered likely but not proven [21].

Animal studies have shown greater increases in muscle mass and fiber CSA with combined low-current electrical stimulation + BFR than with either modality alone, with improved activation of anabolic signaling pathways [22]. Preclinical models offer some preliminary support for combination usage. However, human clinical trials are still rare and inconsistent. While some randomized controlled trials (RCTs) show modest or inconsistent strength benefits and no discernible benefit of combined therapy across outcomes, others show that adding NMES to low-load BFR resistance training results in greater gains in quadriceps activation and CSA compared with BFR alone [20,23,24,25]. In disuse models, combined treatments have reduced lean-mass loss during short-term unloading; however, this has not always resulted in preserved peak force over the same intervals [24]. Only a few studies have provided functional and patient-centered outcomes (pain, performance, neuromuscular activity), and when they do, the findings originate from isolated trials in particular clinical groups with different methods, which restricts generalizability [25].

In conclusion, combined BFR + NMES therapies provide a strong theoretical foundation and some encouraging preclinical and early clinical data for improving muscle activation and reducing disuse-related atrophy. Effects on human strength and hypertrophy, however, remain uneven and appear to be heavily influenced by demographic factors, BFR and stimulation settings, and outcome selection. Firm conclusions on the synergistic superiority of combined BFR + NMES over BFR or NMES alone are not currently supported by the evidence base due to the limited number of high-quality, directly compared trials and the considerable procedural heterogeneity [21,24].

This systematic review and meta-analysis compared combined BFR + NMES with BFR alone for muscle strength, muscle morphology (muscle mass, muscle thickness, and CSA), neuromuscular activation, and functional performance. Meta-analysis was undertaken only when outcomes, measurement units, time points, and study designs were sufficiently comparable and the effect estimates were statistically independent; otherwise, findings were synthesized narratively [26].

## 2. Materials and Methods

### 2.1. Protocol and Registration

This systematic review and meta-analysis was developed a priori and reported in accordance with the Preferred Reporting Items for Systematic Reviews and Meta-Analyses (PRISMA) 2020 statement [26]. The protocol was registered in the International Prospective Register of Systematic Reviews (PROSPERO; CRD420251076162). Eligibility criteria, outcomes, and planned analytical approaches were specified before the quantitative synthesis to reduce selective reporting and analytical flexibility.

### 2.2. Eligibility Criteria

Study eligibility was determined according to predefined criteria structured using the Population, Intervention, Comparator, Outcomes, and Study Design (PICOS) framework.

#### 2.2.1. Population

Eligible studies included human participants of any age, sex, or ethnicity undergoing musculoskeletal rehabilitation. Populations included knee osteoarthritis (KOA), post-anterior cruciate ligament reconstruction, postoperative or injury-related muscle weakness, and muscle disuse atrophy including sarcopenia in older adults. Eligibility was extended to studies conducted in healthy subjects or professional athletes. Exclusions were participants with systemic diseases unrelated to musculoskeletal function, such as cancer or neurological disorders.

#### 2.2.2. Intervention

Eligible interventions were combinations of blood flow restriction (BFR) and neuromuscular electrical stimulation (NMES), compared with BFR alone. To maximize the sensitivity of the search and capture variations in terminology used to describe electrical stimulation, the search strategy incorporated broader terms, including electrical muscle stimulation (EMS), functional electrical stimulation (FES), and related stimulation terminology. During study selection, however, eligibility was determined according to the predefined intervention criteria, and only studies in which the electrical stimulation intervention was consistent with NMES were included.

#### 2.2.3. Comparator

Eligible studies had to include a comparator group receiving BFR training alone, without the addition of EMS. BFR-only interventions had to be performed with standardized or clearly described methods, e.g., pneumatic cuffs or elastic bands, so as to isolate the additive effect of EMS. Comparator groups combining BFR with other active interventions (e.g., nutritional supplementation, cryotherapy, or pharmacologic agents) were excluded unless the independent effect of BFR alone could be clearly isolated.

#### 2.2.4. Outcomes

The prespecified primary outcomes were muscle strength and muscle morphology, including muscle mass, muscle thickness, and CSA. Secondary outcomes included neuromuscular activation, muscle fatigue, and functional performance, including CMJ performance. Outcomes were considered separately when they differed materially in measurement unit, physiological construct, or assessment time point.

Outcomes were synthesized quantitatively when at least two studies reported sufficiently comparable data. Outcomes that could not be pooled because of incompatible measures, time points, or insufficient numerical data were summarized narratively.

#### 2.2.5. Study Design

Only RCTs were eligible, including parallel-group, crossover, and within-participant limb-randomized trials. Non-randomized studies, observational designs, case reports, case series, reviews, conference abstracts, study protocols, animal studies, and in vitro studies were excluded.

### 2.3. Information Sources and Search Strategy

A comprehensive and systematic literature search was conducted to identify eligible randomized controlled trials evaluating the combined effects of BFR and electrical muscle stimulation interventions. We searched MEDLINE via PubMed, Scopus, and the Cochrane Central Register of Controlled Trials (CENTRAL) from inception through 15 September 2026. A complementary search of Google Scholar was also conducted on 15 September 2026. There were no restrictions on publication date, and only English-language studies were eligible.

The search strategy included a combination of controlled vocabulary terms (including Medical Subject Headings (MeSH)) and free-text keywords related to BFR and electrical muscle stimulation interventions. Search concepts were combined using Boolean operators (AND/OR), and database-specific adaptations were applied where appropriate.

For MEDLINE via PubMed, we used the following search strategy: (“blood flow restriction”[tiab] OR BFR[tiab] OR “vascular occlusion”[tiab] OR “occlusion training”[tiab] OR KAATSU[tiab] OR “tourniquet training”[tiab] OR “blood flow restriction training”[tiab] OR “Blood Flow Restriction Training”[Mesh]) AND (“electrical muscle stimulation”[tiab] OR “neuromuscular electrical stimulation”[tiab] OR NMES[tiab] OR “functional electrical stimulation”[tiab] OR FES[tiab] OR “Russian stimulation”[tiab] OR “electrostimulation”[tiab] OR “Electric Stimulation Therapy”[Mesh] OR “Electric Stimulation”[Mesh]) AND (randomized[tiab] OR randomised[tiab] OR “randomized controlled trial”[pt] OR “controlled clinical trial”[pt]) AND (English[lang]) NOT (animals[MeSH] NOT humans[MeSH]). This search yielded 23 results.

For Scopus, the search strategy was: TITLE-ABS-KEY (“blood flow restriction” OR BFR OR “occlusion training” OR “vascular occlusion” OR kaatsu OR “blood flow occlusion”) AND TITLE-ABS-KEY (“electrical muscle stimulation” OR EMS OR “neuromuscular electrical stimulation” OR NMES OR “functional electrical stimulation” OR FES OR “Russian stimulation” OR electrostimulation OR electrotherapy) AND TITLE-ABS-KEY (“muscle strength” OR “muscle hypertrophy” OR “functional performance” OR “muscle function” OR rehabilitation), which yielded 49 results.

For the Cochrane Central Register of Controlled Trials, the search strategy was: (“blood flow restriction” OR BFR OR “vascular occlusion” OR “occlusion training” OR KAATSU OR “tourniquet training” OR “blood flow restriction training”) AND (“electrical muscle stimulation” OR “neuromuscular electrical stimulation” OR NMES OR “functional electrical stimulation” OR FES OR “Russian stimulation” OR electrostimulation), which yielded 41 results.

A supplementary search was also performed in Google Scholar using the keywords (“blood flow restriction” OR “occlusion training” OR KAATSU) AND (“electrical muscle stimulation” OR “neuromuscular electrical stimulation” OR NMES OR EMS) combined with strength, hypertrophy, or rehabilitation. The first 100 results were screened for potentially relevant studies.

To ensure comprehensive coverage, the search was further supplemented by manual screening of the reference lists of included studies and relevant review articles, as well as forward and backward citation tracking. When necessary, corresponding authors or subject-matter experts were contacted to identify additional potentially eligible studies. The search and reporting procedures followed the recommendations of the PRISMA 2020 Statement.

### 2.4. Study Selection Process

All retrieved records were imported into reference management software, and duplicates were removed. Titles and abstracts were screened independently by two reviewers using the pre-specified criteria for eligibility. Studies judged by either reviewer to be potentially relevant for the review were retrieved in full text for assessment.

The full-text articles were independently assessed by the same reviewers for final inclusion. Disagreements were discussed, and a third reviewer was consulted if consensus could not be reached. The process of study selection was depicted with a PRISMA flow diagram.

During full-text assessment, each report was evaluated against the prespecified eligibility criteria, including the eligible population, intervention and comparator requirements, outcome domains, and randomized controlled trial design. Reports were excluded when they did not meet the required outcome criteria, did not use an eligible study design, were protocols or unfinished studies without completed trial results, or did not satisfy the prespecified language or publication eligibility requirements. Screening decisions were made independently by two reviewers, with disagreements resolved through discussion and, when necessary, consultation with a third reviewer.

### 2.5. Data Extraction

Data extraction was undertaken independently by two reviewers using a standardized and piloted data extraction form to ensure consistency and to minimize risk of bias. The extraction form was pre-tested on a sub-sample of included studies before data extraction.

Disputes between reviewers were resolved by discussion or, if necessary, by a third reviewer. When information required for quantitative synthesis was unavailable, contacting study authors was considered as a potential means of obtaining the missing data. However, author contact was not required for the quantitative analyses in this review, as the necessary information was available from the published reports. The extracted data were saved in a structured database.

### 2.6. Data Items

Extracted data included study characteristics (authors, year, country, design, randomization unit, and analysis unit), participant characteristics, sample size, intervention and comparator protocols, outcomes and assessment time points, change-score or endpoint means and variances, paired effect estimates or within-participant correlations where applicable, adherence, withdrawals, adverse events, statistical methods, funding, and conflicts of interest.

For each included study, intervention characteristics were examined for exercise intensity and prescription (including relative load where reported), training volume, treatment frequency, intervention duration, BFR pressure and application schedule, and NMES stimulation parameters. The availability of these details in the published reports was also considered when assessing the comparability of the BFR + NMES and BFR-alone groups and the suitability of studies for quantitative synthesis.

### 2.7. Risk of Bias Assessment

Two reviewers independently assessed the risk of bias of included RCTs using the Cochrane Risk of Bias 2 (RoB 2) tool. The evaluation was based on five domains: bias arising from the randomization process; bias due to deviations from intended interventions; bias due to missing outcome data; bias in measurement of outcomes; and bias in selection of the reported result.

Risk of bias judgments were made by domain and at the outcome level where applicable, rated as low risk, some concerns, or high risk of bias. A global risk of bias judgment was then assigned to each study. Disagreements between reviewers were resolved by consensus or by the opinion of a third reviewer.

### 2.8. Reporting Bias Assessment

Formal funnel-plot assessment and statistical tests for small-study effects were not performed because fewer than 10 studies contributed to the meta-analysis. With so few studies, these methods would have very low power and could be misleading. The possibility of missing results and publication bias was therefore considered qualitatively when interpreting the certainty and completeness of the evidence.

Searches of multiple bibliographic sources were supplemented by reference-list screening and citation tracking. Nevertheless, the small evidence base and restriction to English-language reports limited the ability to exclude publication and language bias.

### 2.9. Certainty of Evidence

The certainty of evidence for each primary and secondary outcome was assessed using GRADE (Grading of Recommendations Assessment, Development and Evaluation) [27]. Because the included studies were RCTs, the certainty of the evidence was initially rated high and was downgraded when concerns were identified for risk of bias, inconsistency, indirectness, imprecision, or publication bias.

GRADE assessment was performed independently by two reviewers, and disagreements were resolved by discussion and consensus. Summary of Findings tables were created to report pooled estimates with their certainty ratings.

### 2.10. Data Synthesis and Statistical Analysis

For continuous outcomes, mean differences (MDs) were calculated from reported change-from-baseline means and standard deviations. No change-score standard deviation was imputed. The MD was defined as the mean change in the BFR-alone group minus the mean change in the BFR + NMES group; therefore, negative values favour BFR + NMES. Because the included studies reported rectus femoris (RF) electromyographic root mean square (EMG RMS) outcomes on different numerical scales, standardized mean differences were calculated using Hedges’ g. Effects were calculated as BFR alone minus BFR + NMES; therefore, negative values favour BFR + NMES. Study-level effect estimates and 95% confidence intervals reported in this review were independently recalculated from the published change-score means, standard deviations, and analysed sample sizes. Consequently, these review-derived estimates may differ from the between-group statistical tests reported in the primary trials.

Clinical and methodological comparability was assessed separately for each outcome before quantitative synthesis. The assessment considered participant population and clinical or experimental context; exercise modality, intensity and prescription, and training volume where reported; treatment frequency and intervention duration; BFR pressure prescription, cuff characteristics, and application schedule; NMES parameters; and outcome definition, measurement method, assessment timing, and study design. The reported protocols were examined to determine whether the BFR + NMES and BFR-alone groups received comparable exercise interventions and whether differences in treatment exposure could affect interpretation of the estimated incremental effect of NMES. When intervention characteristics were incompletely reported, comparability could not be confirmed for those characteristics. Quantitative pooling was based on the overall clinical and methodological comparability of the studies, not solely on whether they reported the same broad outcome.

CSA and EMG RMS were synthesized narratively rather than pooled because only two trials reported each outcome and the trials differed in participant population, intervention duration and, for EMG, signal processing and normalization. For transparency, exploratory pooled estimates for these two outcomes are provided in Supplementary File S3.

The torque-based synthesis was restricted to the two independent parallel-group training trials by Li et al. and Yang et al. These studies shared several intervention characteristics, including multiweek low-load squat training at 25% of 1RM, similar circumference-based BFR pressure prescriptions, nominal NMES settings of 75 Hz, 400 μs and 50 mA with a 3-s ON/3-s OFF cycle, and assessment of knee-extensor peak torque. The experimental limb-unloading study by Slysz et al. was excluded from this torque synthesis because it addressed muscle responses during disuse rather than adaptations during active training; its findings were retained in the narrative synthesis. The torque synthesis was therefore considered exploratory.

Study estimates were pooled using a restricted maximum-likelihood random-effects model with Hartung–Knapp confidence intervals. Statistical heterogeneity was described using τ^2^, Cochran’s Q and I^2^. Given the small number of studies per outcome, heterogeneity statistics were interpreted descriptively. No formal subgroup analysis or meta-regression was undertaken to identify the sources of inconsistency. Calculations were reproduced using Python version 3.12.13 (Python Software Foundation, Beaverton, OR, USA), with NumPy version 2.3.5 and SciPy version 1.17.0 (open-source scientific computing projects affiliated with NumFOCUS, Austin, TX, USA).

For crossover and within-participant limb-randomized trials, quantitative inclusion required a paired MDs with its standard error or standard deviation, or sufficient information to derive it. Neither Slysz and Burr [23] nor Santiago-Pescador et al. [28] reported the covariance or paired variance required for the review comparison. No within-participant correlation was therefore imputed, and these studies were synthesized narratively. Santiago-Pescador et al. [28] reported RF and vastus lateralis (VL) thickness in the same participants; both sites were described narratively and were not treated as independent effects. Each study contributed no more than one independent estimate to any meta-analysis.

The quantitative analyses were conducted in R version 4.5.3 (R Foundation for Statistical Computing, Vienna, Austria) using the *meta* package. For continuous outcomes measured using the same scale and units, effects were expressed as mean differences (MDs), whereas standardized mean differences were used when studies assessed the same underlying construct using different measurement scales. For the torque-based strength analysis, a random-effects meta-analysis was conducted using the restricted maximum-likelihood (REML) estimator for between-study variance and the Hartung–Knapp method for confidence intervals. The analysis was implemented using the ‘metacont()’ function, with group sample sizes, means, and standard deviations entered for the BFR-alone and BFR + NMES groups. The extracted study-level numerical inputs used in the quantitative synthesis are provided in Supplementary File S2, including the group-level data required to examine the reported effect estimates.

### 2.11. Ethics and Dissemination

This study synthesized data from previously published trials and did not involve the collection of individual participant data. Therefore, ethical approval and informed consent were not required.

The findings are reported in accordance with PRISMA 2020 (Table S1) and are intended for dissemination through peer-reviewed publication and scientific presentation.

## 3. Results

### 3.1. Study Selection

Database searches (MEDLINE via PubMed, Scopus, and the Cochrane Central Register of Controlled Trials) and a supplementary Google Scholar search identified 213 records (MEDLINE via PubMed, *n* = 23; Scopus, *n* = 49; Cochrane CENTRAL, *n* = 41; Google Scholar, *n* = 100). After removal of 66 duplicates, 147 records underwent title and abstract screening, of which 121 were excluded. Of the 26 reports sought for retrieval, 4 could not be retrieved, leaving 22 reports for full-text eligibility assessment. Seventeen reports were excluded at this stage: 5 did not report eligible outcomes, 2 did not meet the required study-design criteria, 7 were protocols or unfinished studies without completed trial results, 1 was published in Japanese and therefore did not meet the language eligibility criterion, and 2 were classified as grey-literature records. These decisions were made in relation to the prespecified eligibility criteria described in Section 2.2. The remaining 5 studies were included in the review (Figure 1).

### 3.2. Study Characteristics

Five randomized trials involving 174 participants met the eligibility criteria [23,24,25,28,29]. Three were parallel-group trials (134 participants), one was a crossover trial (20 participants), and one randomized 40 limbs within 20 participants. Table 1 summarizes their designs, participant characteristics, intervention protocols, and outcomes. The studies represented distinct contexts: healthy participants undergoing active training [23,25], healthy participants exposed to experimental limb unloading [24], an acute crossover experiment in healthy sedentary adults [28], and low-load training in male football players with knee osteoarthritis [29]. These contexts differ in their physiological aims and should not automatically be treated as interchangeable when interpreting treatment effects.

The intervention characteristics summarized in Table 2 were also considered when assessing exercise-dose comparability. In the two parallel-group training trials contributing to the quantitative synthesis, Li et al. [25] and Yang et al. [29] prescribed low-load squat training at 25% of 1RM, but the intervention lasted six weeks with five sessions per week in Li et al. [25] and eight weeks with four sessions per week in Yang et al. [29] Thus, the nominal relative exercise load was similar, whereas treatment frequency and intervention duration differed. Similarity in these characteristics did not establish equivalence in total training volume or overall treatment exposure. The available intervention descriptions were therefore interpreted alongside the differences in population and outcome measurement when deciding whether quantitative pooling was appropriate.

### 3.3. Risk of Bias

The Cochrane RoB 2 method was used to evaluate the risk of bias in five areas: the randomization procedure, changes from planned interventions, missing outcome data, outcome measurement, and choice of reported result. No trial was deemed to have an overall high risk of bias; all five included trials were considered to raise “some concerns”, primarily due to the randomization method and outcome assessment domains (Figure 2).

### 3.4. Narrative Synthesis

Numerical results for outcomes synthesized narratively rather than pooled quantitatively, including group-level results and between-group estimates where reported, are provided in Table 3.

#### 3.4.1. Muscle Strength

Five studies evaluated muscle strength, although the designs, intervention durations, and strength metrics differed substantially [23,24,25,28,29]. Slysz and Burr [23] and Santiago-Pescador et al. [28] reported isometric knee-extension strength in kilograms, whereas Slysz et al. [24], Li et al. [25], and Yang et al. [29] assessed knee-extensor torque in newton-metres. Multi-week training studies generally reported larger within-group gains with BFR + NMES, but these findings were not consistent across short-term disuse and acute-fatigue models. In the 14-day unloading study by Slysz et al. [24], strength declined similarly across groups despite preservation of muscle mass in the combined-intervention group. In the acute crossover study by Santiago-Pescador et al. [28], transient fatigue-related reductions occurred without a clear between-condition advantage. The marked clinical and temporal heterogeneity therefore limits any general conclusion that adding NMES consistently improves strength.

#### 3.4.2. Muscle Mass

Four trials in all assessed how BFR + NMES affected muscle mass. Two of these studies used Dual-energy X-ray absorptiometry (DEXA) to evaluate whole-thigh lean mass (g) [23,24], while Li et al. [25] and Yang et al. [29] assessed rectus femoris cross-sectional area (CSA) using ultrasonography. These CSA outcomes are described in Section 3.4.6. After six weeks of intervention, Slysz and Burr [23] found no statistically significant difference in leg muscle size between intervention groups, although the change scores showed a trend toward a group difference (*p* = 0.07). Using a predefined increase of more than 1 g in muscle mass to classify positive responders, 70% of participants in the combined BFR + NMES group met this criterion, compared with 60% in the NMES-alone group, 30% in the BFR-alone group, and 20% in the control group. These responder proportions were descriptive and did not establish a statistically significant between-group difference in muscle size. In the 14-day unloading study by Slysz et al. [24], whole-thigh lean mass decreased by 4% ± 1% in the control group (*p* < 0.001) and 3% ± 2% in the BFR-alone group (*p* = 0.001), whereas the BFR + EMS group showed a mean change of −0.3% ± 3% (*p* = 0.8). These *p*-values describe within-group changes from baseline. The numerical pattern suggests that combining BFR with EMS may attenuate lean-mass loss during short-term disuse; however, a claim of statistically significant superiority over BFR alone should be made only if supported by the study’s direct between-group comparison. Similarly, after 8 weeks of low-load training with BFRT or BFR + NMES, Yang et al. [29] found significant increases in RF CSA in football players with knee OA. The BFR + NMES, BFRT, and EMS groups all showed increases; however, group comparisons revealed that only the BFR + NMES and BFRT groups had significantly higher CSA values than the EMS and control groups. The BFR + NMES and BFRT groups did not differ significantly, suggesting that adding EMS to BFR did not further promote muscle growth. Across the multiweek training studies, BFR-containing interventions were associated with improvements in muscle morphology. In their original analyses, neither Li et al. nor Yang et al. reported a statistically significant difference in rectus femoris CSA between BFR + NMES and BFR alone (*p* = 0.916 and *p* = 0.525, respectively). However, the review-derived change-score estimate for Yang et al. favoured BFR + NMES and excluded the null, whereas the estimate for Li et al. did not. The possible attenuation of lean-mass loss during short-term experimental disuse observed by Slysz et al. is preliminary and should not be generalized to active training or clinical rehabilitation populations. It also does not establish that a possible effect on muscle mass translates into preserved strength or improved functional performance.

#### 3.4.3. Muscle Thickness

Two studies examined the effect of BFR + NMES on muscle thickness, with Slysz et al. [24] focusing only on the VL, and Santiago-Pescador et al. [28] examining both the RF and VL. The two studies in question differed in purpose and timescale. For example, Slysz et al. [24] evaluated changes in VL thickness (cm) over a 14-day unloading period, and Santiago-Pescador et al. [28] measured acute RF and VL muscle thickness (mm) changes in a single 25-min session. In the Slysz et al. [24] disuse model, VL muscle thickness significantly decreased during the 2-week unloading period in the control group, but not in the BFR + NMES or BFR groups. Moreover, muscle thickness changes differed between groups, with a significantly more favorable outcome in the BFR + NMES condition compared with BFR alone (*p* = 0.02), suggesting that the addition of NMES may offer further protection against muscle atrophy during prolonged immobilization. Additionally, Santiago-Pescador et al. [28] found that rectus femoris muscle thickness was greater under both combined BFR + NMES and BFR alone than under NMES alone immediately after the intervention and 15 min later. Combined BFR + NMES was not significantly superior to BFR alone, and no significant between-condition differences were observed for vastus lateralis thickness. These acute responses diminished within 15 min and should be interpreted as transient muscle swelling rather than structural hypertrophy. Overall, since neither study demonstrates chronic hypertrophic changes in thickness, these outcomes should not be interpreted as direct measures of long-term muscle growth.

#### 3.4.4. Countermovement Jump (CMJ)

The effects of combining BFR and NMES on CMJ performance and fatigue-related outcomes were investigated in two studies [28,29]. Because both studies evaluated CMJ performance as a measure of neuromuscular exhaustion or lower-limb explosive performance, they were included in the qualitative synthesis. Santiago-Pescador et al. [28] measured CMJ performance prior to and immediately after the intervention and analyzed the acute effects of BFR plus NMES under various stimulation conditions. All conditions showed a substantial drop in CMJ performance from PRE to POST; however, there were no significant differences between the intervention groups, suggesting that the protocol caused fatigue independent of the intervention used. In contrast, Yang et al. [29] assessed CMJ height in football players with KOA after an 8-week training session. Both the BFRT and EMS groups and the combined BFR + NMES group showed within-group increases in CMJ height; however, the combined group showed significantly greater improvements than the BFRT, EMS, and control groups, while both BFRT and EMS alone showed greater improvements than the control group. Overall, the multi-week intervention reported in Yang et al. [29] showed improvements in CMJ height following training, with the combined BFR + NMES intervention producing the greatest improvement in jump performance, whereas the acute protocol in Santiago-Pescador et al. [28] showed reductions in CMJ performance consistent with fatigue across all conditions.

#### 3.4.5. Muscle Activation (EMG RMS)

Two parallel-group studies investigated rectus femoris surface EMG RMS following multiweek training [25,29]. Both studies assessed the same broad muscle and outcome domain. Li et al. reported a study-level estimate of −1.24 (95% CI −2.20 to −0.28), whereas Yang et al. reported an estimate of −4.49 (95% CI −5.79 to −3.19). However, the studies differed in participant population, intervention duration, and aspects of EMG processing and normalization. These differences limit confidence in the magnitude of a common effect. Because only two studies contributed and their estimates differed substantially in magnitude, these findings should not be taken as definitive evidence of a consistent additional NMES effect on muscle activation. The exploratory pooled estimate is provided in Supplementary File S3 (Figure S2).

#### 3.4.6. Cross-Sectional Area (CSA)

Two studies reported rectus femoris morphology following multiweek training [25,29]. Li et al. [25] studied healthy young men following six weeks of low-load squat training, whereas Yang et al. [29] studied male university football players with knee osteoarthritis after eight weeks. Both studies labelled their reported morphology outcome as rectus femoris CSA and assessed changes following training. The studies differed in participant population and intervention duration; these differences may contribute to differences between the study findings and limit generalizability, so the results are presented for each study separately.

The original trial analyses reported no statistically significant difference in CSA between BFR + NMES and BFR alone in either Li et al. (*p* = 0.916) or Yang et al. (*p* = 0.525). In contrast, the review-derived change-score estimate for Yang et al. favoured BFR + NMES and excluded the null (MD −0.83 cm^2^, 95% CI −1.31 to −0.36), whereas the estimate for Li et al. did not (MD −0.08 cm^2^, 95% CI −0.31 to 0.15). With only two studies, the reasons for the difference between these estimates cannot be reliably evaluated. The exploratory pooled estimate is provided in Supplementary File S3 (Figures S1 and S2).

### 3.5. Quantitative Synthesis (Meta-Analysis)

#### Muscle Strength—Torque-Based (Nm)

Two independent parallel-group trials involving 52 participants contributed to this exploratory torque-based analysis [25,29]. Li et al. [25] and Yang et al. [29] shared several intervention characteristics, including multiweek low-load squat training at 25% of 1RM, similar circumference-based BFR pressure prescriptions, nominal NMES settings of 75 Hz, 400 μs and 50 mA with a 3-s ON/3-s OFF cycle, and assessment of knee-extensor peak torque. These similarities provided a rationale for examining the studies together. However, the populations, intervention duration, and strength-testing procedures differed, so the pooled estimate should not be interpreted as a definitive common treatment effect.

The review-derived study-level mean differences favoured BFR + NMES in both trials: −71.00 Nm for Li et al. [25] (95% CI −94.67 to −47.33) and −15.08 Nm for Yang et al. [29] (95% CI −19.51 to −10.65). However, the pooled change-score estimate was highly imprecise and crossed the null (MD −41.78 Nm, 95% CI −396.68 to 313.12; *p* = 0.375). Substantial statistical heterogeneity was observed (I^2^ = 95.2%; Q = 20.72, df = 1, *p* < 0.0001). Although both study-level estimates favoured BFR + NMES under the direction convention used in this review, the pooled confidence interval was extremely wide and included effects in either direction. Therefore, this exploratory analysis does not establish that adding NMES improves torque-based strength, and the magnitude of any additional effect remains highly uncertain. The study-level and pooled estimates are presented in Figure 3.

The experimental limb-unloading study by Slysz et al. [24] was excluded from this torque synthesis because it addressed responses during disuse rather than adaptations during active training. Its findings remain in the narrative synthesis: knee-extensor strength declined similarly across groups despite the numerical preservation of thigh lean mass observed in the BFR + EMS group.

### 3.6. Certainty of the Evidence (GRADE)

Using the GRADE approach, the certainty of evidence for CSA was judged to be very low, mainly due to methodological limitations across included trials, inconsistent study-level results, and imprecision given the small number of studies and participants. The certainty of evidence for muscle activation (EMG RMS) was judged to be very low owing to methodological limitations, large differences in the magnitude of study-level effects, and imprecision given the small number of studies and participants. The certainty of evidence for CMJ was judged very low because the evidence came from one parallel-group trial and one crossover trial addressing different physiological contexts, and the paired variance required for pooling was unavailable. The certainty of evidence for load-based strength was judged very low because both contributing studies used dependent designs, paired variances were unavailable, and the results were imprecise and clinically heterogeneous. The certainty of evidence for muscle thickness was judged very low because the two studies evaluated different contexts, the crossover study reported two correlated muscle sites, and the covariance required for quantitative synthesis was unavailable. The certainty of evidence for torque-based strength outcomes (Nm) was judged to be very low owing to methodological limitations, substantial heterogeneity (I^2^ = 95.2%), and imprecision. The torque evidence was downgraded one level for inconsistency because heterogeneity was substantial (I^2^ = 95.2%) and the study-level effect estimates differed markedly. No separate downgrade was applied for indirectness because both studies directly addressed the eligible intervention comparison and prespecified torque outcome. The certainty of evidence for muscle mass was judged very low because of risk-of-bias concerns, indirectness because the evidence was driven by one small experimental-disuse trial, and serious imprecision. The within-limb study could not be pooled without covariance information, while the parallel disuse trial suggested possible preservation of thigh lean mass with BFR + NMES. The certainty-of-evidence assessments and reasons for downgrading are summarized in Table 4.

Only torque-based strength was quantitatively synthesized, and this estimate was interpreted as exploratory because of the small number of studies and substantial uncertainty surrounding the pooled effect. CSA and EMG RMS were each reported by only two studies and were therefore described narratively. The torque analysis was restricted to the two active-training trials, but its confidence interval remained extremely wide and statistical heterogeneity was substantial. Heterogeneity statistics were interpreted descriptively, and the available evidence did not permit reliable formal investigation of the sources of inconsistency. Certainty of evidence was therefore low or very low across outcomes.

## 4. Discussion

### 4.1. Summary of Main Findings

This review evaluated the incremental effect of adding NMES to BFR across five small randomized trials. The studies addressed different clinical and physiological contexts, including active training in healthy participants, training in football players with knee osteoarthritis, acute physiological responses, and experimental limb unloading. These contexts should not be considered interchangeable.

Only torque-based strength was quantitatively synthesized, and this pooled estimate requires cautious interpretation. CSA and EMG RMS were each reported by two studies whose populations, intervention durations, and measurement methods differed, limiting confidence in a common effect magnitude. The torque analysis was restricted to two active-training trials with similar intervention protocols, but its confidence interval was extremely wide and heterogeneity remained substantial. Overall, the evidence does not establish a consistent additional benefit of BFR + NMES over BFR alone. The apparent preservation of muscle mass during short-term experimental disuse is preliminary and should not be generalized to active training or clinical rehabilitation populations.

Accordingly, the absence of a statistically clear pooled benefit should not be interpreted as proof that the interventions are equivalent, because the available studies are too few and the estimates too imprecise to exclude potentially meaningful benefits or harms.

### 4.2. Muscle Strength: A Benefit That Depends on Context

The five trials assessed strength using different measures and in different physiological contexts. Li et al. and Yang et al. assessed knee-extensor peak torque following multiweek low-load training, whereas Slysz et al. evaluated torque during experimental limb unloading. Li and Yang shared several intervention characteristics and provided the basis for the exploratory torque analysis. However, their populations, training durations, and strength-testing procedures differed. The pooled estimate (MD −41.78 Nm, 95% CI −396.68 to 313.12; *p* = 0.375) was extremely imprecise, and statistical inconsistency was substantial (I^2^ = 95.2%). The result should not be interpreted as establishing a common or clinically generalizable effect.

The Slysz et al. disuse study remains in the narrative synthesis rather than being pooled with active-training studies. Although BFR + EMS showed a numerical pattern consistent with preservation of thigh lean mass, knee-extensor strength declined similarly across groups. This finding illustrates that an apparent effect on muscle mass does not necessarily translate into preserved strength and that evidence from experimental disuse cannot be assumed to represent the response to active training or postoperative rehabilitation.

### 4.3. Muscle Morphology: Does NMES Add Anything to BFR-Induced Hypertrophy?

The included studies assessed muscle morphology using different methods and in different contexts. The Slysz studies assessed whole-thigh lean mass, and Slysz et al. also measured vastus lateralis thickness during experimental disuse. Santiago-Pescador et al. measured acute changes in muscle thickness after a single intervention session; these transient changes should not be interpreted as evidence of chronic hypertrophy.

Li et al. and Yang et al. reported rectus femoris morphology following six and eight weeks of training, respectively. Both labelled their reported outcome as CSA and used ultrasound-based assessment. The studies differed in population and intervention duration, and their study-level CSA estimates differed (MD −0.08 cm^2^ for Li et al. and −0.83 cm^2^ for Yang et al.). These findings should therefore be interpreted cautiously and not as definitive evidence of an additional hypertrophic benefit from NMES.

Although the original analyses by Li et al. and Yang et al. did not report statistically significant CSA differences between BFR + NMES and BFR alone, the review-derived change-score estimate for Yang et al. excluded the null, whereas the estimate for Li et al. did not. This difference reflects the distinction between the trials’ reported statistical tests and the review’s independently recalculated study-level contrasts. Given the discordant study-level estimates and the inclusion of only two studies, the findings do not establish a consistent additional effect of NMES on muscle morphology. The potential preservation of muscle mass during short-term experimental disuse remains based on one small study and does not establish a general effect during active training or across rehabilitation populations.

### 4.4. Neuromuscular Activation and Functional Performance

Both studies assessed rectus femoris EMG RMS following multiweek training. However, the study-level estimates differed substantially (−1.24 and −4.49), and differences in participant population, intervention duration, EMG processing, and normalization limit confidence in the magnitude of a common effect. With only two studies, the source of the discrepancy cannot be reliably investigated. These findings should therefore be interpreted cautiously and do not establish a dependable additional effect of NMES on muscle activation.

Functional outcomes require the same context-specific interpretation. Santiago-Pescador et al. assessed immediate fatigue-related responses after an acute session, whereas Yang et al. assessed adaptation after eight weeks of training. These are different physiological questions, so their CMJ findings were appropriately synthesized narratively.

### 4.5. Situating These Findings Within the Broader Literature

Only one other systematic review has quantitatively synthesized combined BFR + NMES, and it was published while the present review was underway. Mangahas et al. [30] pooled three trials, including two also included here [23,24], plus one additional soleus study, and compared combined BFR + NMES against a broader “CONTROL” condition (BFR alone, NMES alone, or no intervention combined), reporting a medium but non-significant pooled effect for skeletal muscle hypertrophy (d = 0.61–0.64, 95% CI crossing zero). Despite a largely non-overlapping analytic approach (standardized MDs pooled via a multilevel model, versus MDs pooled by outcome here) and a different comparator definition, the two reviews converge on the same qualitative conclusion: a directionally positive but statistically fragile signal for a hypertrophic benefit of adding NMES to BFR. This review extends that picture in two directions. First, it deals with the more clinically actionable question that faces a clinician who has already decided to use BFR: is it worth adding NMES? The comparator is limited specifically to BFR alone, rather than the broader question of whether the combination beats doing nothing. Second, our review includes Li et al. [25], a trial published in 2023 that was not included in Mangahas et al.’s review because it combined BFR and electrical muscle stimulation with low-intensity squat training, which fell outside their eligibility criteria excluding combined interventions involving other exercise methods. Our review also includes Yang et al. [29], which was published in October 2025, after Mangahas et al.’s February 2025 search cutoff. In addition, our review examines strength, neuromuscular activation, and functional outcomes beyond the hypertrophy-focused scope of that review. The more general literatures of BFR alone and NMES alone provide useful bounds for interpreting both reviews. BFR alone without adjunct already elicits strength and hypertrophy adaptations that are comparable to high-load resistance training in many contexts [5,8,9], and NMES alone has an established, though modest, role in alleviating early postoperative quadriceps weakness after knee surgery and anterior cruciate ligament reconstruction [16,17]. Combined BFR + NMES therefore enters a field in which both parent interventions already have real, independently demonstrated effects, which makes isolating an incremental benefit of combining them scientifically important and empirically difficult, precisely the situation reflected in the very small, heterogeneous evidence base synthesized here.

Recent evidence from the broader BFR literature also supports the need to distinguish the effects of BFR itself from any incremental effect of adding NMES. A 2026 systematic review and meta-analysis of musculoskeletal rehabilitation trials found that low-load exercise with BFR produced greater improvements in muscle strength and disability than comparable low-load exercise without BFR, while differences relative to high-load exercise were less consistent and depended on the outcome and certainty of evidence [31]. An umbrella review incorporating 47 previous meta-analyses likewise found that BFR exercise generally improves muscle strength and hypertrophy compared with low-load exercise, although the quality of the underlying review evidence varied from low to moderate [32]. In populations with knee pain, however, recent evidence remains mixed: a 2025 systematic review reported some short-term benefits of BFR-based exercise but emphasized the small size and methodological limitations of the available trials [33]. Similarly, a 2025 meta-analysis focused on KOA found no clear additional benefit of BFR over conventional resistance training for several clinical outcomes, although subgroup analyses suggested possible advantages for muscle size under low-load conditions [34]. These findings reinforce the importance of treating BFR as an active intervention in its own right and of using a BFR-only comparator when attempting to determine whether NMES provides an additional therapeutic effect.

Evidence from postoperative and sports-medicine populations provides a similar context. Recent systematic reviews of BFR after anterior cruciate ligament reconstruction have reported improvements in selected measures of muscle strength and patient-reported recovery, although results for muscle volume, pain, and other functional outcomes remain variable and the certainty of evidence is frequently limited by heterogeneity and methodological concerns [35,36]. A further review examining BFR before and after ACL reconstruction reported improvements in postoperative isokinetic strength and quadriceps CSA but no consistent improvement in knee-related function, again with very low certainty of evidence [37]. These findings are relevant to the present review because they illustrate that BFR may have clinically meaningful effects in rehabilitation even when its effects on individual structural and functional outcomes are not uniform.

These reviews address BFR or NMES as separate interventions and therefore provide only indirect context for the present question. None of the five included trials directly evaluated BFR + NMES after ACL reconstruction; the effectiveness of the combined intervention in this population remains undetermined.

### 4.6. Certainty of the Evidence and Clinical Implications

The certainty of evidence was low or very low across outcomes. Interpretation was limited by methodological concerns, small samples, indirectness for specific rehabilitation populations, and imprecision. The torque-based strength estimate was retained as an exploratory quantitative synthesis, but it does not establish a reliable additional benefit of NMES over BFR alone. Only two studies contributed to each of the CSA and EMG outcomes, which were therefore described narratively, and the torque analysis retained substantial statistical inconsistency despite restricting the comparison to two active-training trials.

The findings should not be interpreted as establishing that BFR + NMES is superior to BFR alone across musculoskeletal rehabilitation populations. In particular, findings from healthy participants, football players with knee osteoarthritis, and individuals undergoing experimental limb unloading are not interchangeable. A possible effect on thigh lean mass during short-term experimental disuse is preliminary and does not demonstrate a corresponding improvement in strength or functional performance. None of the included trials directly investigated BFR + NMES after ACL reconstruction; its effectiveness in this population remains undetermined.

Clinically, the true effect could differ substantially in either direction from the estimates reported here, and combined BFR + NMES should not yet be treated as an evidence-based upgrade over BFR alone. Both interventions have generally reassuring safety records when appropriately applied, but BFR should be prescribed with attention to individual vascular characteristics, medical history, cuff selection, and the degree of arterial occlusion [38,39]. Contemporary literature increasingly emphasizes individualized pressure prescription based on arterial or limb occlusion pressure rather than reliance on a single fixed cuff pressure, because cuff width, limb morphology, vascular characteristics, body position, and device characteristics can substantially influence the delivered stimulus [38]. Risk-stratification frameworks have also emphasized careful screening for cardiovascular, thrombotic, and other clinical risk factors before BFR is applied in rehabilitation settings [39]. A supervised trial of the combination may be most defensible when voluntary muscle activation is markedly limited, such as early after major joint surgery, during immobilization, or with arthrogenic muscle inhibition, but this remains a hypothesis rather than an established indication.

The clinical rationale for NMES is also supported by evidence from rehabilitation populations outside the present BFR-specific comparison. A systematic review in healthy adults found that electrical stimulation can improve muscle strength, but substantial variation in stimulation intensity, frequency, duration, electrode placement, and training context prevented identification of a single optimal protocol [40]. In patients undergoing TKA, a meta-analysis of nine RCTs involving 691 participants reported improvements in quadriceps strength and selected measures of pain and physical function following postoperative NMES, although not all outcomes reached clinically meaningful thresholds [41]. More recently, a systematic review focused on ACL surgery similarly reported beneficial effects of NMES on quadriceps strength and selected measures of knee function [42]. A 2024 scoping review further highlighted the potential role of NMES during periods of limb immobilization, while also emphasizing substantial variation in clinical application and limited high-quality evidence [43]. Collectively, these findings support the biological and clinical plausibility of NMES in settings of impaired voluntary activation, while also demonstrating why stimulation parameters and rehabilitation context should be carefully considered when interpreting the present findings. However, these findings concern BFR or NMES as separate interventions and provide only indirect context for the present review. None of the five included trials directly evaluated combined BFR + NMES following ACL reconstruction; therefore, the effectiveness of the combined intervention in this population remains undetermined.

### 4.7. Strengths and Limitations

Strengths of this review include PROSPERO registration, independent duplicate screening and extraction, risk-of-bias assessment using RoB 2, and GRADE assessment [26,27]. Nevertheless, the evidence base comprised only five small randomized trials, involving 174 participants in total, and the quantitative synthesis included only two studies. Consequently, the pooled estimate is vulnerable to imprecision and between-study heterogeneity, and the evidence is insufficient to support firm conclusions about the additional effects of NMES when combined with BFR. The trials differed in participant population, clinical or experimental context, BFR pressure and prescription method, NMES parameters, intervention duration, and outcome-assessment procedures. Although intervention characteristics were extracted and summarized in Table 2, the available reports did not establish that exercise intensity, total training volume, and overall treatment exposure were equivalent between the BFR + NMES and BFR-alone groups across all included studies. In the two multiweek training trials contributing to the quantitative synthesis, the nominal exercise load was similar, but treatment frequency and intervention duration differed. Consequently, the pooled estimate cannot be interpreted as isolating the effect of adding NMES under otherwise identical exercise-dose conditions. Incomplete reporting and the small number of studies also prevented reliable investigation of the independent contributions of these protocol differences to the observed heterogeneity.

The robustness of the quantitative findings also warrants consideration. Although alternative meta-analytic approaches may provide useful information, we did not conduct sensitivity analyses using alternative statistical methods. Consequently, we cannot determine whether the pooled estimate would remain similar under different analytical approaches. This limitation is particularly relevant given the small number of studies contributing to the pooled outcome and the substantial heterogeneity observed. The pooled estimate should therefore be interpreted cautiously as an exploratory finding rather than definitive evidence of an additional benefit of NMES when combined with BFR. Further studies with larger samples and more comparable intervention and outcome-assessment protocols are needed to confirm these findings.

The torque-based analysis was retained because the contributing studies shared broad intervention characteristics that provided a rationale for quantitative comparison. Nevertheless, it remains exploratory, as the estimate is highly imprecise and statistically inconsistent despite restricting the comparison to two active-training studies. CSA and EMG RMS were each reported by only two studies that differed in population and intervention duration (and, for EMG, in processing and normalization), and were therefore synthesized narratively. The small number of studies precluded reliable investigation of the sources of heterogeneity. Accordingly, heterogeneity statistics were interpreted descriptively rather than used to establish specific causes of inconsistency. The present review focused on the incremental effect of adding NMES to BFR and was not designed to estimate the independent effectiveness of NMES alone. Although some included trials contained additional intervention arms, the independent effects of NMES were not synthesized as a separate research question. Future systematic reviews could compare NMES alone, BFR alone, and their combination to clarify the relative and incremental contributions of each intervention.

The review process also had limitations. Only English-language reports were eligible; one Japanese report was excluded, two grey-literature records were excluded, and four potentially relevant reports could not be retrieved. Google Scholar screening was limited to the first 100 results, and trial registries were not reported as separate search sources. In addition, the very small number of studies precluded reliable assessment of small-study effects and limited formal exploration of heterogeneity. Additionally, the Scopus search included an additional outcome-related term block intended to focus retrieval on studies addressing clinically relevant muscle and functional outcomes. However, requiring these terms may have reduced search sensitivity by excluding otherwise eligible studies that did not explicitly use the specified terminology in their titles, abstracts, or keywords. This possibility should be considered when interpreting the comprehensiveness of the literature search. Additionally, the individual reports excluded following full-text assessment and their corresponding reasons for exclusion were not listed in the manuscript or Supplementary Materials, which represents a limitation in the report-level transparency of the study-selection process. These limitations may have increased the risk of missing studies or results and should be considered when interpreting the findings.

The revised analysis addressed unit-of-analysis concerns by excluding crossover and within-participant limb-randomized estimates from pooling when paired variance or covariance information was unavailable. Multiple muscle-site outcomes from the same Santiago-Pescador participants were not entered as independent estimates. This avoids double-counting but reduces statistical power and highlights the need for primary studies to report paired effect estimates and covariance information.

Protocol heterogeneity is not unique to the combined intervention literature. Recent reviews of BFR and electrical stimulation have similarly identified substantial variation in pressure prescription, cuff characteristics, stimulation parameters, exercise loading, treatment frequency, and outcome assessment, limiting direct comparison between studies [40,44]. Recent NMES research has additionally demonstrated that differences in stimulation strategy can influence evoked torque, fatigability, discomfort, and strength adaptations, further supporting the need for standardized reporting of stimulation parameters in future BFR + NMES trials [44].

### 4.8. Future Research Directions

Progress in this area is likely to depend less on additional small exploratory trials and more on a small number of adequately powered, methodologically standardized studies. Priorities include: individualizing BFR pressure to a percentage of limb occlusion pressure rather than a fixed cuff pressure, and prescribing NMES intensity as a percentage of MVC rather than “maximum tolerable” intensity, so that findings across future trials become directly comparable rather than confounded by dosing; adopting a core outcome set for combined BFR + NMES trials, since outcome heterogeneity has now limited quantitative synthesis in both this review and the only other existing meta-analysis on the topic [30]; recruiting clinically relevant populations—patients early after TKA or anterior cruciate ligament reconstruction, and older adults with sarcopenia—rather than predominantly healthy young or athletic volunteers; and extending follow-up beyond eight weeks to determine whether any early advantage of adding NMES persists, plateaus, or is overtaken by BFR alone once voluntary training has had time to act. Given that the possible muscle-mass preservation finding in this review arose from a single experimental disuse model, a dedicated, adequately powered trial of combined BFR + NMES during a defined period of post-surgical or post-injury immobilization—where the mechanistic rationale for NMES is most compelling—represents a particularly high-value next step.

The choice of study population also warrants greater attention. BFR has been investigated in older adults with sarcopenia, where low-load exercise may provide a practical alternative when conventional high-load resistance exercise is difficult to tolerate [37]. However, the existing evidence remains insufficient to determine whether the addition of NMES provides meaningful advantages in older or clinically compromised populations. Future trials should therefore prioritize populations in whom voluntary activation or loading capacity is genuinely constrained, rather than relying predominantly on healthy young participants, and should evaluate patient-centered outcomes alongside laboratory measures of muscle morphology and activation.

## 5. Conclusions

Current evidence does not establish a consistent additional benefit of adding NMES to BFR. The included trials differed in participant populations, clinical and experimental contexts, intervention protocols, and outcome measurement. The torque-based strength meta-analysis was retained as an exploratory quantitative synthesis, but its small evidence base, substantial heterogeneity, and imprecision, together with the limited and inconsistent narrative findings for CSA and EMG RMS, prevent firm conclusions about the magnitude or generalizability of any additional NMES effect. A possible effect on preserving muscle mass during short-term experimental disuse remains preliminary and should not be generalized to active training or clinical rehabilitation. None of the included trials directly evaluated BFR + NMES after anterior cruciate ligament reconstruction; therefore, its effectiveness in this population remains undetermined. Overall, the available evidence is insufficient to determine whether adding NMES to BFR provides a clinically meaningful additional benefit, and the findings should be interpreted as preliminary rather than as evidence of equivalence or absence of an effect.

## Figures and Tables

**Figure 1:**
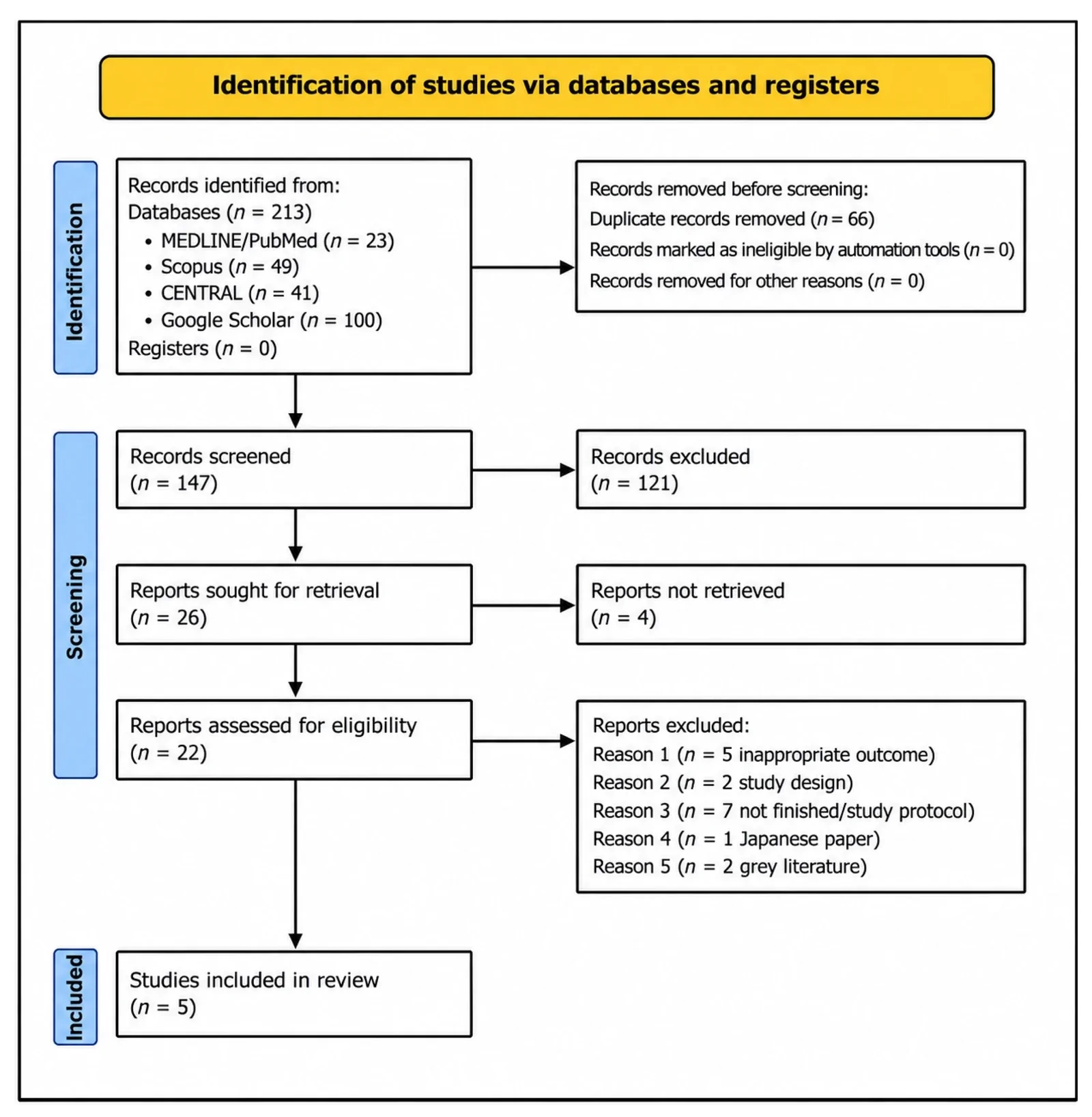
PRISMA 2020 flow diagram of study identification, screening, and inclusion.

**Figure 2:**
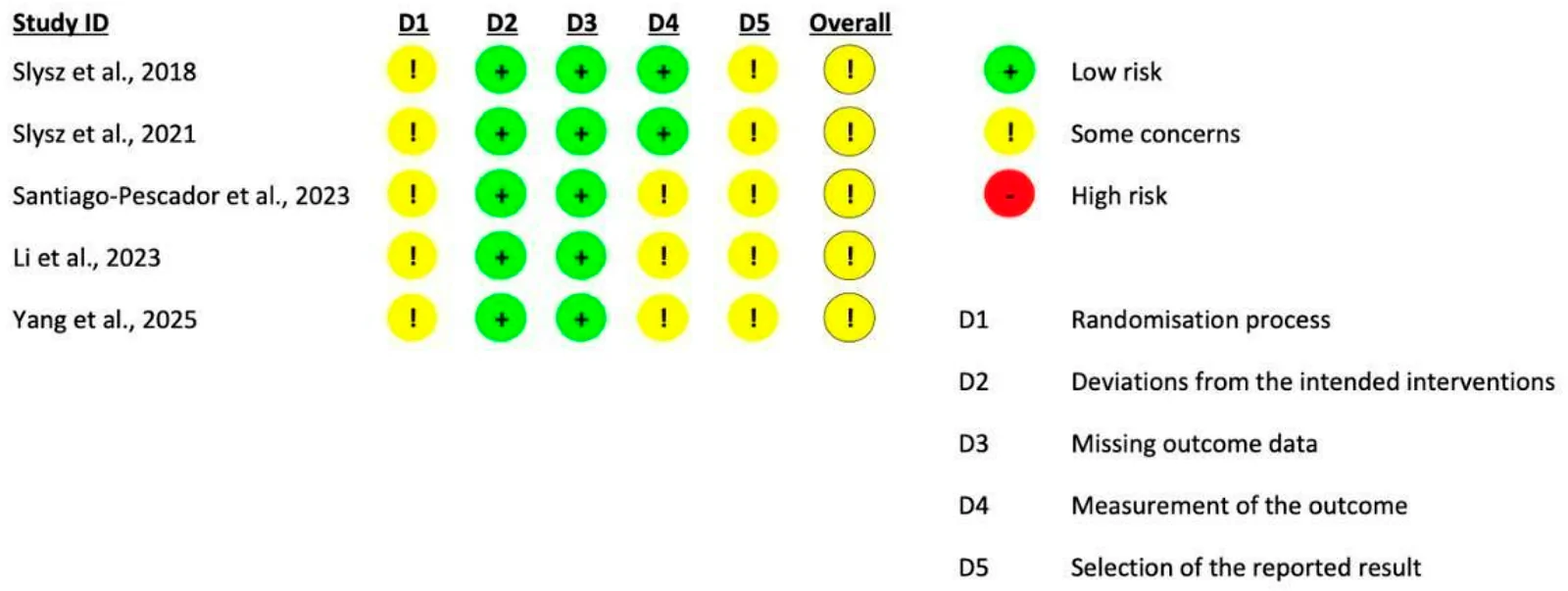
Risk-of-bias (RoB 2) judgments for each domain in each included RCTs. Included studies are cited as: Slysz et al. (2018) [23], Li et al. (2023) [25], Santiago-Pescador et al. (2023) [28], Slysz et al. (2021) [24], and Yang et al. (2025) [29].

**Figure 3:**
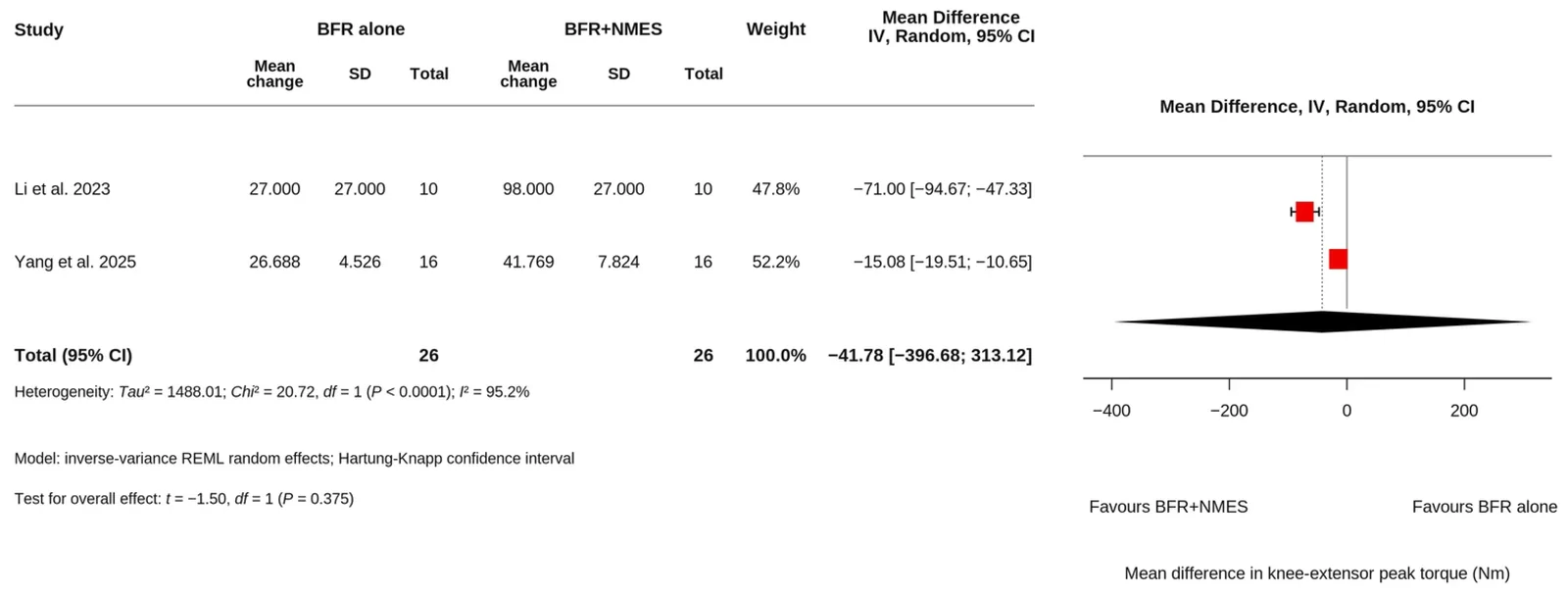
Random-effects meta-analysis of change in knee-extensor peak torque (Nm), calculated as the mean change in the BFR-alone group minus the mean change in the BFR + NMES group. Negative values favour BFR + NMES. Mean and SD columns show change from baseline, and Total represents the number of participants analysed in each group. The analysis includes two parallel-group active-training trials and should be interpreted cautiously because of the substantial heterogeneity and extremely wide pooled confidence interval. The red color of the study-specific markers is used for aesthetic purposes only and does not represent any additional information. Included studies are cited as: Li et al. [25] and Yang et al. [29].

**Table 1:** Characteristics and key findings of included studies. Abbreviations: BFR, blood flow restriction; BFRT, blood flow restriction training; CSA, cross-sectional area; CMJ, countermovement jump; DEXA, dual-energy X-ray absorptiometry; EBRT, elastic band resistance training; EMG, electromyography; EMS, electrical muscle stimulation; MVC, maximal voluntary contraction; NMES, neuromuscular electrical stimulation; PT, peak torque; RF, rectus femoris; RMS, root mean square; VAS, visual analogue scale; VL, vastus lateralis.

Author Year	Study Design	Objective	Participants	NMES Protocol	BFR Protocol	Outcomes	Follow-Up	Primary Finding
Slysz JT, Burr JF, 2018 [23]	Randomized within-participant limb trial (40 limbs; 10 limbs per condition)	To examine the independent and combined effects of blood flow restriction (BFR) and low-intensity neuromuscular electrical stimulation (NMES) on skeletal muscle strength and hypertrophy	20 recreationally active adults (40 randomized limbs; 10 limbs allocated to each of four conditions)	Quadriceps stimulation; pulse width 400 μs; frequency 50–100 Hz; applied at maximally tolerable intensity; continuous during sessions	Automated tourniquet; 220 mmHg; 3 cycles per session of 4 min inflation + 4 min deflation; cuff placed proximal on the thigh	(1) Quadriceps isometric strength (kg) (2) Thigh muscle mass (DEXA, g)	Pre-intervention and post-intervention (6 weeks; assessed 24–72 h after final session)	Combined BFR + NMES significantly increased knee extensor strength compared with control, but no significant hypertrophy was observed due to high inter-individual variability
Slysz JT, Boston M, King R, Pignanelli C, Power GA, Burr JF, 2021 [24]	Randomized controlled trial during experimental limb disuse	To determine whether combining BFR with electrical muscle stimulation (EMS) attenuates loss of muscle mass and strength during a period of limb unloading	30 healthy young adults (14 males, 16 females); mean age ≈ 22 years	Quadriceps EMS; duty cycle 6 s ON/15 s OFF; pulse width 200 μs; frequency 60 Hz; intensity set to elicit 15% MVC; applied continuously during BFR	Full arterial occlusion using personalized limb occlusion pressure (≈180–290 mmHg); 3 cycles of 5 min occlusion + 5 min reperfusion; twice daily, 5 days/week	(1) Whole-thigh lean mass (DEXA) (2) VL thickness (ultrasound) (3) Knee extension MVC (Nm)	Baseline and post-disuse (14 days of unloading)	BFR + EMS completely preserved thigh muscle mass during disuse, whereas BFR alone did not; strength declined similarly in all groups
Li N, Yang J, Liao Y, 2023 [25]	Randomized controlled trial (4-arm parallel design)	To examine the effects of BFRT, EMS, and their combination on muscle activation, hypertrophy, and strength during low-intensity squat training.	40 healthy young men (≈20–21 years), physically healthy, low physical activity levels, naïve to BFRT/EMS/squat training	Biphasic stimulation; 75 Hz, 400 μs pulse width, 50 mA intensity; duty cycle 3 s ON/3 s OFF; 3 × 5-min stimulation bouts per session	Pneumatic cuff (5 cm width) placed at groin; pressure individualized by thigh circumference (200–350 mmHg); 3 cycles of 5 min inflation + 2 min deflation per session	(1) RF cross-sectional area (CSA) (2) EMG RMS (muscle activation) (3) knee extensor peak torque (PT)	Pre- and post-intervention only (6-week intervention)	Combined BFR + NMES significantly improved muscle strength and activation compared with BFRT alone and control, indicating synergistic benefits for neuromuscular adaptation
Santiago-Pescador S, Fajardo-Blanco D, López-Ortiz S, et al., 2023 [27]	Randomized crossover acute experimental study	To examine acute effects of NMES, BFR, and combined BFR + NMES on muscle thickness (cell swelling) and fatigue in the lower limbs	20 healthy, young, sedentary adults (15 women, 5 men; mean age ≈ 25 years)	Biphasic current; low frequency (10 Hz) and high frequency (80 Hz); pulse width 350 μs; current set to tolerable intensity (≈7/10 VAS); 8 s ON/3 s OFF for 25 min	Thigh cuff inflated to 50% of individually measured arterial occlusion pressure (mean AOP 181.8 ± 26.0 mmHg); continuous during 25-min intervention	(1) RF and VL muscle thickness (ultrasound) (2) countermovement jump (CMJ) (3) maximal voluntary contraction (MVC)	Immediate, post-intervention, and 15 min post	Rectus femoris muscle thickness was greater under both combined BFR + NMES and BFR alone than under NMES alone immediately after the intervention and 15 min later. Combined BFR + NMES was not significantly superior to BFR alone. No significant between-condition differences were observed for vastus lateralis thickness or fatigue.
Yang J, Li N, He S, Peng X, Yang J, Chen J, Zheng Y, Zou Y, Liao Y, 2025 [28]	Parallel-group randomized controlled trial	To investigate whether combining BFRT with EMS improves muscle strength, muscle activation, muscle volume, and sports performance in football players with knee osteoarthritis	64 male university football players with knee osteoarthritis (16 participants per group)	Quadriceps EMS; 75 Hz; pulse width 400 μs; intensity 50 mA; duty cycle 3 s ON/3 s OFF; applied during squat training	Pneumatic cuff (5 cm width) at groin; pressure based on thigh circumference (200–350 mmHg); 3 cycles of 5 min inflation + 2 min deflation per session	(1) Muscle strength (peak torque) (2) Muscle activation (EMG RMS) (3) Muscle volume (RF CSA) (4) Sports performance (10 m sprint, 20 m sprint, CMJ, Illinois agility test)	Baseline and post-intervention (8 weeks)	BFRT combined with EMS produced the greatest improvements in muscle strength, muscle volume, neuromuscular activation, and sprint, jump, and agility performance compared with BFRT or EMS alone

**Table 2:** Characteristics of the Blood Flow Restriction and Neuromuscular Electrical Stimulation Interventions in the Included Studies. Abbreviations: AOP, arterial occlusion pressure; MVC, maximal voluntary contraction; NIRS, near-infrared spectroscopy; VAS, visual analogue scale; 1RM, one-repetition maximum. EMS is the terminology used in some source papers; BFR + NMES is the umbrella term used in this review.

Study	BFR Prescription	NMES Prescription	Treatment Schedule	Contribution to Meta Analysis
Slysz and Burr [23]	Fixed 220 mmHg; approximately 10.2-cm cuff individualized to leg size; full restriction confirmed using NIRS; 3 cycles of 4-min inflation and 4-min deflation	50–100 Hz; reported pulse-train duration 400 μs; maximally tolerable intensity; quadriceps electrodes targeting vastus lateralis and vastus medialis	Four days/week for six weeks; intervention sessions during the training period	Not included in meta analysis
Slysz et al. [24]	Individualized full arterial occlusion pressure, approximately 180–290 mmHg; 3 cycles of 5-min occlusion separated by 5-min reperfusion	60 Hz; 200 μs; intensity targeting knee-extensor torque equivalent to 15% MVC; 6-s ON/15-s OFF	BFR + EMS during experimental limb unloading; twice daily, five days/week for 14 days	Not included in meta analysis
Li et al. [25]	5-cm cuff at groin; pressure determined by thigh circumference: 200, 250, 300 or 350 mmHg; 3 cycles of 5-min inflation and 2-min deflation	75 Hz; 400 μs; 50 mA; 3-s ON/3-s OFF	Low-load squat training at 25% 1RM, five days/week for six weeks	Included in meta analysis
Santiago-Pescador et al. [28]	Wide thigh cuff; applied pressure at 50% of individually measured AOP; mean AOP 181.8 ± 26.0 mmHg	10-Hz and 80-Hz stimulation conditions; 350 μs; individually tolerable intensity, approximately 7/10 on VAS; 8-s ON/3-s OFF	Acute 25-min intervention; outcomes assessed immediately and, for muscle thickness, after 15 min	Not included in meta analysis
Yang et al. [29]	5-cm cuff at groin; pressure determined by thigh circumference: 200, 250, 300 or 350 mmHg; 3 cycles of 5-min inflation and 2-min deflation	75 Hz; 400 μs; 50 mA; 3-s ON/3-s OFF	Low-load squat training at 25% 1RM, four days/week for eight weeks	Included in meta analysis

**Table 3:** Caption.Narrative synthesis of study-level results excluded from quantitative pooling. Abbreviations: ANCOVA, analysis of covariance; BFR, blood flow restriction; BFRT, blood flow restriction training; CI, confidence interval; CMJ, countermovement jump; COMB, combined BFR + NMES; CON, control; DEXA, dual-energy X-ray absorptiometry; EMS, electrical muscle stimulation; ES, effect size; MD, mean difference; MVC, maximal voluntary contraction; NMES, neuromuscular electrical stimulation; Nm, newton-metre; PRE/POST, before/after the intervention; RCT, randomized controlled trial; RF, rectus femoris; SD, standard deviation; VL, vastus lateralis. EMS is the terminology used in some source papers; BFR + NMES is the umbrella term used in this review.

Outcome	Study (Design; Participants)	Measure and Time Point	Results Reported For Each Group	BFR + NMES vs. BFR Alone (Between-Group Result)	Reason Not Pooled
Muscle strength	Slysz and Burr [23]; randomized within-participant limb trial; 20 participants recruited, with 19 completing the 6-week intervention (38 limbs analyzed).	Isometric quadriceps strength (kg); change from baseline to 6 weeks.	BFR + NMES (COMB) increased strength by 32 ± 19 kg, significantly more than control (3 ± 11 kg; post-hoc *p* = 0.02). BFR alone: +18 ± 17 kg; NMES alone: +16 ± 28 kg (neither differed significantly from control). Responders (>1 kg increase): COMB 100%, NMES 100%, BFR 60%, control 30%.	The paper reports that BFR + NMES was not significantly different from BFR alone. An exact mean difference with 95% CI or pairwise *p*-value is not reported in the available text.	Within-participant limb randomization; paired variance/covariance not reported
	Slysz et al. [24]; parallel-group RCT during experimental limb unloading; 30 participants (control, BFR, BFR + EMS)	Knee-extension MVC (Nm); baseline to 14 days of unloading	Strength declined similarly across all groups. Group means ± SD: CON: −32 ± 23 Nm (−18%); BFR: −17 ± 17 Nm (−10%); BFR + EMS: −24 ± 20 Nm (−18%).	The study’s overall between-group comparison was not statistically significant (ANCOVA: F = 0.94, *p* = 0.40). An exact pairwise mean difference with 95% CI or pairwise *p*-value is not reported in the strength results.	Disuse model rather than active training; single parallel trial in this context
	Santiago-Pescador et al. [28]; randomized crossover, acute; 20 participants	Isometric knee-extension strength/MVC (kg); before vs. after a single 25-min session	Significant pre-to-post decrease with both BFR + NMES conditions and BFR alone (*p* ≤ 0.022); no significant change with NMES alone (*p* ≥ 0.510). Isometric knee-extension MVC (kg), pre → post, mean ± SD: BFR + NMES, high-frequency current: 67.75 ± 21.73 → 62.77 ± 19.62 (ES −0.24) BFR + NMES, low-frequency current: 68.27 ± 23.20 → 65.88 ± 22.71 (ES −0.10) BFR alone: 67.12 ± 22.22 → 64.44 ± 20.33 (ES −0.13)	No significant difference between conditions (*p* ≥ 0.258 for the condition effect; time × condition *p* = 0.002). Pairwise *p*-value or MD (95% CI) for BFR + NMES vs. BFR alone not reported.	Crossover design; paired variance not reported
Muscle mass (DEXA)	Slysz and Burr [23]; randomized within-participant limb trial; 20 participants (40 limbs; 10 limbs per condition)	Thigh muscle mass (DEXA, g); baseline to 6 weeks	Proportion of responders (gain > 1 g; descriptive): BFR + NMES 70%; NMES alone 60%; BFR alone 30%; control 20%.	Group difference in change scores not statistically significant (*p* = 0.07, as reported). Mean thigh muscle mass (g), pre → post (SD of change not reported): BFR + NMES 9139 → 9234 (+95); BFR alone 9313 → 9418 (+105); NMES alone 9311 → 9391 (+80); control 7385 → 7384 (−1). Pairwise BFR + NMES vs. BFR alone not reported.	Within-participant limb randomization; covariance not reported
	Slysz et al. [24]; parallel-group RCT during experimental limb unloading; 30 participants (control, BFR, BFR + EMS)	Whole-thigh lean mass (DEXA); % change from baseline to 14 days of unloading	BFR + NMES: −0.3 ± 3% (within-group *p* = 0.8) BFR alone: −3 ± 2% (*p* = 0.001) Control: −4 ± 1% (*p* < 0.001)	Group × time interaction *p* = 0.004; ANCOVA on change F = 6.88, *p* = 0.004. BFR + NMES differed from BFR alone at *p* < 0.05. BBFR alone vs. control: *p* = 0.1. Crude difference in mean % change (BFR alone − BFR + NMES) = −2.7 percentage points (derived from the group means above; CI not calculable because group sizes and the type of dispersion are not stated); negative favours BFR + NMES.	Only one parallel trial; disuse context not comparable with the within-limb training trial
Muscle thickness (ultrasound)	Slysz et al. [24]; parallel-group RCT during experimental limb unloading; 30 participants (control, BFR, BFR + EMS)	Vastus lateralis thickness (cm); baseline to 14 days of unloading	Control: significant decrease. BFR and BFR + NMES: no significant decrease. Change in VL thickness (cm), mean ± SD: control −0.19 ± 0.12 (−7%; *p* = 0.005); BFR alone −0.19 ± 0.26 (*p* = 0.07); BFR + NMES +0.09 ± 0.22 (*p* = 0.07). *n* = 10 per group.	Change differed between groups, more favourable with BFR + NMES than BFR alone (ANCOVA *p* = 0.03; pairwise *p* = 0.02). Crude difference in mean change (BFR alone − BFR + NMES) = −0.28 cm, 95% CI −0.51 to −0.05 (derived from the reported means, SDs and *n* = 10 per group; unadjusted; negative favours BFR + NMES).	Different time scale and context (14-day disuse vs. single session); crossover covariance unavailable
	Santiago-Pescador et al. [28]; randomized crossover, acute; 20 participants	Rectus femoris and vastus lateralis thickness (mm); immediately after and 15 min after a single 25-min session	Thickness increased immediately after the intervention in all conditions; the change was largely gone by 15 min (acute swelling). Mean ± SD, mm (PRE → POST → POST 15 min): RF, BFR + NMES high-frequency: 17.37 ± 3.13 → 19.27 ± 2.91 (+10.9%, ES 0.63) → 18.27 ± 3.02 (+5.2%) RF, BFR + NMES low-frequency: 17.62 ± 3.22 → 19.32 ± 3.09 (+9.6%, ES 0.54) → 18.21 ± 2.78 (+3.3%) RF, BFR alone: 17.72 ± 3.12 → 19.20 ± 3.09 (+8.4%, ES 0.47) → 18.33 ± 3.22 (+3.4%) VL, BFR + NMES high-frequency: 22.34 ± 3.57 → 23.95 ± 3.82 → 22.81 ± 3.63 VL, BFR + NMES low-frequency: 22.41 ± 3.87 → 23.66 ± 4.09 → 22.85 ± 3.84 VL, BFR alone: 22.42 ± 4.01 → 23.56 ± 3.69 → 22.75 ± 3.71	Rectus femoris thickness was greater under both combined BFR + NMES and BFR alone than under NMES alone at POST (*p* ≤ 0.018) and POST 15 min (*p* ≤ 0.016). Combined BFR + NMES was not significantly superior to BFR alone. For vastus lateralis thickness, neither the condition effect (*p* = 0.732) nor the time × condition interaction (*p* = 0.322) was significant.	Crossover design; two correlated muscle sites; covariance not reported
Countermovement jump (CMJ)	Santiago-Pescador et al. [28]; randomized crossover, acute; 20 participants	CMJ performance; before vs. after a single 25-min session	Significant decrease from pre to post in all five conditions (*p* ≤ 0.022). CMJ height (cm), pre → post, mean ± SD: BFR + NMES, high-frequency: 13.67 ± 3.85 → 11.62 ± 3.52 (ES −0.56) BFR + NMES, low-frequency: 13.79 ± 4.23 → 12.15 ± 3.56 (ES −0.42) BFR alone: 13.51 ± 4.08 → 12.32 ± 3.59 (ES −0.31)	No significant difference between conditions (*p* ≥ 0.258; time × condition *p* = 0.002). Pairwise *p*-value or MD (95% CI) for BFR + NMES vs. BFR alone not reported. Crude difference in mean change (BFR alone − BFR + NMES): −1.19 − (−2.05) = +0.86 cm (high-frequency) and −1.19 − (−1.64) = +0.45 cm (low-frequency); derived, CI not calculable.	Acute fatigue vs. training adaptation; crossover paired variance not reported
	Yang et al. [29]; parallel-group RCT, 4 arms; 64 football players with knee osteoarthritis (16 per group)	CMJ height; baseline to 8 weeks	Within-group increases in BFRT, EMS and BFR + NMES groups (all *p* < 0.001); no change in control. CMJ height, mean ± SD (units not stated in the paper), pre → post (change); *n* = 16 per group: BFR + NMES: 36.386 ± 1.849 → 40.740 ± 2.444 (+4.354 ± 1.232) BFRT alone: 35.912 ± 2.366 → 38.509 ± 2.647 (+2.596 ± 0.886) EMS alone: 34.910 ± 2.211 → 37.363 ± 2.374 (+2.453 ± 0.874) Control: 35.043 ± 1.747 → 34.984 ± 1.766 (−0.058 ± 0.588)	Post-intervention CMJ significantly higher with BFR + NMES than with BFRT alone (*p* = 0.019; ES −0.876, 95% CI of ES −1.597 to −0.142), and than EMS (*p* < 0.001) and control (*p* < 0.001). No baseline differences. Crude difference in mean post-intervention value (BFRT alone − BFR + NMES) = −2.23, 95% CI −4.07 to −0.39 (derived from the reported means, SDs and *n* = 16; reproduces the reported *p* = 0.019); difference in mean change = −1.76, 95% CI −2.53 to −0.98 (derived). Negative favours BFR + NMES.	Different context from the acute trial; paired variance unavailable for the crossover trial

**Table 4:** Summary of findings (GRADE) for BFR + NMES versus BFR alone. Abbreviations: CI, confidence interval; CSA, cross-sectional area; EMG, electromyography; GRADE, Grading of Recommendations Assessment, Development and Evaluation; I^2^, I-squared statistic; MD, mean difference; Nm, Newton-metre; RCT, randomized controlled trial; RoB 2, Cochrane Risk of Bias 2; RMS, root mean square.

Outcome	Evidence InCluded	Effect or Synthesis Result	Certainty (GRADE)	Reasons for Rating	Domain-Specific Downgrades (Levels)
Cross-sectional area (CSA)	2 parallel RCTs (52 participants); narrative synthesis	Not pooled: study-level MDs −0.08 cm^2^ (Li et al.) and −0.83 cm^2^ (Yang et al.)	Very low	Methodological limitations, inconsistency between the two studies, and serious imprecision	Risk of bias −1; inconsistency −1; indirectness 0; imprecision −1; publication bias 0. Total: −3 levels.
Muscle activation (rectus femoris EMG RMS)	2 parallel RCTs (52 participants); narrative synthesis	Not pooled: study-level Hedges’ g −1.24 (Li et al.) and −4.49 (Yang et al.)	Very low	Processing/normalization differences, substantial inconsistency and very serious imprecision	Risk of bias −1; inconsistency −1; indirectness 0; imprecision −2; publication bias 0. Total: −4 levels.
Countermovement jump	2 trials (52 participants); narrative synthesis	Not pooled: one parallel-group trial and one crossover trial addressed different contexts, and paired variance was unavailable.	Very low	Downgraded for RoB 2 concerns, indirectness/inconsistency between acute and training contexts, and serious imprecision.	Risk of bias −1; inconsistency 0; indirectness −1; imprecision −1; publication bias 0. Total: −3 levels.
Load-based strength (kg)	2 dependent-design trials (40 participants); narrative synthesis	Not pooled: one within-limb trial and one crossover trial did not report the paired variance or covariance required for synthesis.	Very low	Downgraded for RoB 2 concerns, indirectness, and serious imprecision; quantitative synthesis was not possible without unverifiable assumptions.	Risk of bias −1; inconsistency 0; indirectness −1; imprecision −1; publication bias 0. Total: −3 levels.
Muscle thickness	2 trials (40 participants); narrative synthesis	Not pooled: the crossover study reported two correlated muscle sites, and the required covariance was unavailable.	Very low	Downgraded for RoB 2 concerns, indirectness across acute and disuse contexts, and serious imprecision.	Risk of bias −1; inconsistency 0; indirectness −1; imprecision −1; publication bias 0. Total: −3 levels.
Torque-based strength	2 parallel RCTs (52 participants)	MD −41.78 Nm (95% CI −396.68 to 313.12; *p* = 0.375); I^2^ = 95.2%	Very low	Exploratory; downgraded for risk-of-bias concerns, serious inconsistency (I^2^ = 95.2% and markedly different study effects), and serious imprecision.	Risk of bias −1; inconsistency −1; indirectness 0; imprecision −1; publication bias 0. Total: −3 levels.
Muscle mass	2 trials (40 participants); narrative synthesis	Not pooled: the within-limb trial lacked covariance data; one small parallel disuse trial suggested preservation of thigh lean mass.	Very low	Downgraded for risk-of-bias concerns, indirectness because the evidence was driven by one small experimental-disuse trial, and serious imprecision.	Risk of bias −1; inconsistency 0; indirectness −1; imprecision −1; publication bias 0. Total: −3 levels.

## Data Availability

All data arising from this study are included within the article.

## Supplementary Materials

The following supporting information can be downloaded at: https://www.mdpi.com/article/10.3390/jcm15218725/s1, Table S1: PRISMA 2020 Checklist; Table S2: Arm-level data for cross-sectional area, EMG RMS, and knee-extensor peak torque; Supplementary File S3: Exploratory pooled analyses of cross-sectional area and EMG RMS (Figures S1 and S2).

## Abbreviations

The following abbreviations are used in this manuscript:

1RM	One-repetition maximum
AOP	Arterial occlusion pressure
BFR	Blood flow restriction
BFRT	Blood flow restriction training
CI	Confidence interval
CMJ	Countermovement jump
CSA	Cross-sectional area
DEXA	Dual-energy X-ray absorptiometry
EMG	Electromyography
EMS	Electrical muscle stimulation
FES	Functional electrical stimulation
KOA	Knee osteoarthritis
MDs	Mean differences
MeSH	Medical Subject Headings
mTORC1	Mammalian target of rapamycin complex 1
MVC	Maximal voluntary contraction
NIRS	Near-infrared spectroscopy
NMES	Neuromuscular electrical stimulation
PICOS	Population, Intervention, Comparator, Outcomes, and Study Design
PRISMA	Preferred Reporting Items for Systematic Reviews and Meta-Analyses
RCT	Randomized controlled trial
REML	Restricted maximum likelihood
RF	Rectus femoris
RMS	Root mean square
RoB 2	Cochrane Risk of Bias 2
S6K1	S6 kinase 1
SMD	Standardized mean difference
TKA	Total knee arthroplasty
VAS	Visual analog scale
VL	Vastus lateralis
