# Supplementary material for: Effects of Combined Blood Flow Restriction and Neuromuscular Electrical Stimulation Versus Blood Flow Restriction Alone: A Systematic Review and Meta-Analysis
Source: J Clin Med. 2026 Nov 8;15(21):8725. doi: 10.3390/jcm15218725 (PMC13763699; doi:10.3390/jcm15218725)
Supplement: Supplementary file 1 [file jcm-15-08725-s001.zip › jcm-4610062-Supplementary File S3.pdf]

Supplementary File S3: Exploratory Pooled Analyses of Cross-Sectional Area and EMG RMS

Purpose and status

Cross-sectional area (CSA) and rectus femoris EMG RMS were each reported by only two trials, and statistical heterogeneity was very high (I^2 = 87.1% and 93.6%). The main text therefore describes both outcomes narratively (Sections 3.4.5 and 3.4.6) and draws no conclusions from pooled estimates.

This file reports the exploratory pooled analyses that were calculated for these two outcomes so that readers can inspect them. The pooled estimates are not used for inference. The torque-based strength analysis remains in the main text (Section 3.5.1).

Methods for these two analyses

Both analyses used the same random-effects approach as the torque analysis in the main text, with effects calculated as BFR alone minus BFR+NMES, so negative values favour BFR+NMES.

- **Studies:** the two parallel-group training trials, Li et al. [25] and Yang et al. [29] (reference numbers as in the main text). Each contributed one independent estimate per outcome (10 participants per group in Li, 16 per group in Yang; 52 in total).
- **Basis for pooling:** both trials assessed rectus femoris CSA or EMG RMS after multiweek training. They differed in participant population and intervention duration.
- **CSA:** mean difference (cm^2) from reported change-from-baseline means and standard deviations. No change-score standard deviation was imputed.
- **EMG RMS:** Hedges' g, because the studies reported rectus femoris EMG RMS on different numerical scales.
- **Pooling:** restricted maximum-likelihood (REML) random-effects model with Hartung–Knapp confidence intervals.
- **Heterogeneity:** described with τ^2, Cochran's Q and I^2, and interpreted descriptively. No subgroup analysis or meta-regression was undertaken.
- **Software:** R 4.5.3 (*meta* package); calculations reproduced in Python 3.12.13 (NumPy 2.3.5, SciPy 1.17.0).
- **Study-level estimates:** recalculated from the published change-score means, standard deviations and analysed sample sizes, so they may differ from the between-group tests reported in the primary trials.

Results: cross-sectional area (CSA)

The pooled change in rectus femoris CSA showed no clear difference between BFR+NMES and BFR alone (MD −0.43 cm^2, 95% CI −5.17 to 4.32; 2 trials, 52 participants).

Heterogeneity was substantial (I^2 = 87.1%; τ^2 = 0.2450; Q = 7.76, df = 1, p = 0.005). The study-level estimates were −0.83 cm^2 for Yang et al. (46.0% weight) and −0.08 cm^2 for Li et al. (54.0% weight). With only two studies, the source of the inconsistency cannot be reliably evaluated.

The confidence interval is wide and crosses the null. The result should be read as exploratory and not as establishing a common or generalizable additional effect of NMES on CSA.

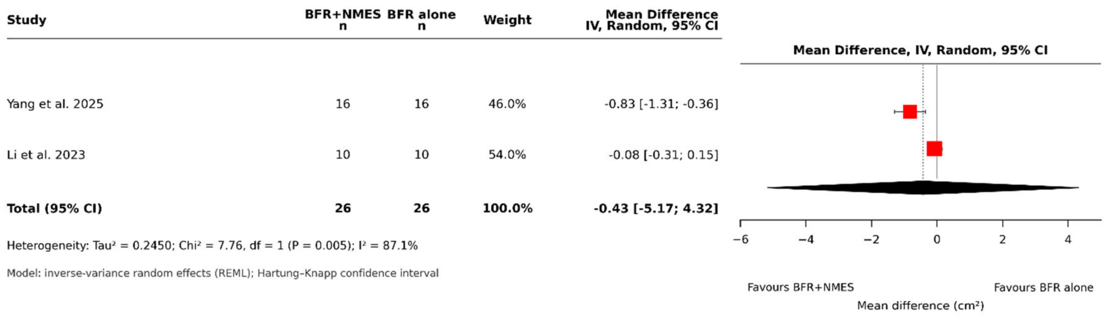

Figure S1. Forest plot of change in rectus femoris CSA (cm²): BFR alone minus BFR+NMES. Negative values favour BFR+NMES. Study-level estimates and confidence intervals were independently recalculated from published change-score summary data and may differ from the statistical tests reported by the primary trials.

Results: EMG RMS

The pooled standardized mean difference in rectus femoris EMG RMS was −2.84 (95% CI −23.47 to 17.80; $p = 0.331$; 2 trials, 52 participants), an extremely imprecise estimate.

Heterogeneity was considerable ($I^2 = 93.6\%$; $\tau^2 = 4.9391$; $Q = 15.55$, df = 1, $p < 0.0001$). The study-level estimates were −1.24 for Li et al. (95% CI −2.20 to −0.28; 51.0% weight) and −4.49 for Yang et al. (95% CI −5.79 to −3.19; 49.0% weight).

Both studies reported rectus femoris EMG RMS after multiweek training, but differences in population, intervention duration, EMG processing and normalization limit confidence in a common effect magnitude. The pooled estimate does not establish a reliable additional effect of NMES on muscle activation.

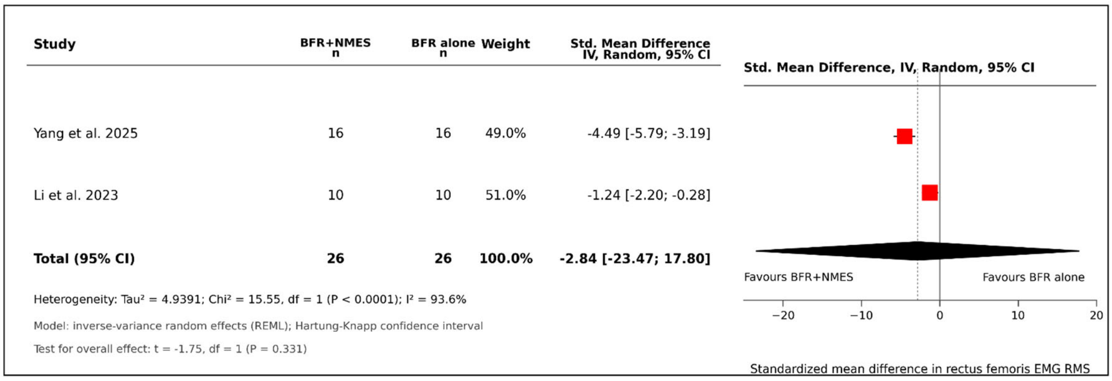

Figure S2. Forest plot of the standardized mean difference in rectus femoris EMG RMS between BFR+NMES and BFR alone. Negative effect estimates favour BFR+NMES. Study-level estimates and confidence intervals were independently recalculated from published change-score summary data.

Interpretation and limitations

These pooled estimates are exploratory and should not be used to judge whether adding NMES to BFR changes CSA or muscle activation.

- **Two studies per outcome:** with 52 participants and two trials, the Hartung–Knapp interval uses one degree of freedom (critical t = 12.71). The pooled intervals are therefore far wider than either study's own interval.
- **Inconsistent magnitude:** the point estimates favoured BFR+NMES in both trials for both outcomes, but the sizes differed (CSA −0.08 vs −0.83 cm^2; EMG RMS −1.24 vs −4.49). I^2 was 87.1% for CSA and 93.6% for EMG RMS.
- **Possible sources:** participant population (healthy young men vs football players with knee osteoarthritis), intervention duration (6 vs 8 weeks) and, for EMG RMS, signal processing and normalization. With two studies these cannot be tested.
- **Recalculated estimates:** the review-derived CSA estimate for Yang et al. excluded the null, whereas in their original analyses neither trial reported a statistically significant CSA difference between BFR+NMES and BFR alone (p = 0.916 for Li et al.; p = 0.525 for Yang et al.).
