# Supplementary material for: Effects of Combined Blood Flow Restriction and Neuromuscular Electrical Stimulation Versus Blood Flow Restriction Alone: A Systematic Review and Meta-Analysis
Source: J Clin Med. 2026 Nov 8;15(21):8725. doi: 10.3390/jcm15218725 (PMC13763699; doi:10.3390/jcm15218725)
Supplement: Supplementary file 1 [file jcm-15-08725-s001.zip › jcm-4610062-Supplementary Table S2 .pdf]

Supplementary Table S2 Arm level data used in the quantitative syntheses

Table S2A Cross-sectional area (cm²)

Study	Muscle	EMS frequency	n BFR	Mean BFR	SD BFR	n BFR+NMES	Mean BFR+NMES	SD BFR+NMES
Yang, 2025 (RF)	Rectus Femoris	75 Hz	16	2.881	0.592	16	3.713	0.769
Li N, 2023 (RF)	Rectus Femoris	75 Hz	10	1.05	0.34	10	1.13	0.16

Table S2B EMG RMS

Study	Muscle	EMS frequency	n BFR	Mean BFR	SD BFR	n BFR+NMES	Mean BFR+NMES	SD BFR+NMES
Yang, 2025 (RF)	Rectus Femoris	75 Hz	16	2.833	3.921	16	20.749	3.856
Li N, 2023 (RF)	Rectus Femoris	75 Hz	10	2.7	4.2	10	8.6	4.5

Table S2C Knee-extensor peak torque (Nm)

Study	Muscle	EMS frequency	n BFR	Mean BFR	SD BFR	n BFR+NMES	Mean BFR+NMES	SD BFR+NMES
Li N, 2023	Knee extensors	75 Hz	10	27	27	10	98	27
Yang, 2025	Knee extensors	75 Hz	16	26.688	4.526	16	41.769	7.824
