# Supplementary material for: Effects of Combined Blood Flow Restriction and Neuromuscular Electrical Stimulation Versus Blood Flow Restriction Alone: A Systematic Review and Meta-Analysis
Source: J Clin Med. 2026 Nov 8;15(21):8725. doi: 10.3390/jcm15218725 (PMC13763699; doi:10.3390/jcm15218725)
Supplement: Supplementary file 1 [file jcm-15-08725-s001.zip › jcm-4610062-Table S1 PRISMA 2020 Checklist.pdf]

PRISMA 2020 Checklist

Section and Topic	Item #	Checklist item	Location where item is reported
**TITLE**			
Title	1	Identify the report as a systematic review.	Reported – manuscript title, p. 1.
**ABSTRACT**			
Abstract	2	See the PRISMA 2020 for Abstracts checklist.	Reported – structured Abstract, p. 1 (Background/Objectives, Methods, Results, Conclusions).
**INTRODUCTION**			
Rationale	3	Describe the rationale for the review in the context of existing knowledge.	Reported – Introduction (section1), pp. 2–4.
Objectives	4	Provide an explicit statement of the objective(s) or question(s) the review addresses.	Reported – Introduction (section 1), final paragraph, p. 4.
**METHODS**			
Eligibility criteria	5	Specify the inclusion and exclusion criteria for the review and how studies were grouped for the syntheses.	Reported – Methods, section 2.2 'Eligibility Criteria' (section 2.2.1–§2.2.5: Population, Intervention, Comparator, Outcomes, Study Design), pp. 4–5.
Information sources	6	Specify all databases, registers, websites, organisations, reference lists and other sources searched or consulted to identify studies. Specify the date when each source was last searched or consulted.	Reported – Methods, section 2.3 'Information Sources and Search Strategy,' p. 5 (databases/sources listed; searched from inception to July 2026).
Search strategy	7	Present the full search strategies for all databases, registers and websites, including any filters and limits used.	Reported – Methods, section 2.3 'Information Sources and Search Strategy,' pp. 5–6 (full Boolean strategies for PubMed, Scopus, Cochrane CENTRAL, and Google Scholar terms, with language filter).
Selection process	8	Specify the methods used to decide whether a study met the inclusion criteria of the review, including how many reviewers screened each record and each report retrieved, whether they worked independently, and if applicable, details of automation tools used in the process.	Reported – Methods, section 2.4 'Study Selection,' p. 6 (2 reviewers, independent screening, third-reviewer arbitration).
Data collection process	9	Specify the methods used to collect data from reports, including how many reviewers collected data from each report, whether they worked independently, any processes for obtaining or confirming data from study investigators, and if applicable, details of automation tools used in the process.	Reported – Methods, section 2.5 'Data Extraction,' p. 6 (2 reviewers, piloted form, third-reviewer arbitration, authors contacted for missing data); automation tools not mentioned.
Data items	10a	List and define all outcomes for which data were sought. Specify whether all results that were compatible with each outcome domain in each study were sought (e.g. for all measures, time points, analyses), and if not, the methods used to decide which results to collect.	Partially reported – Methods, section 2.2.4 'Outcomes' (p. 5) and section 2.6 'Data Items' (p. 6) list outcomes sought, but do not state whether all compatible results (all measures/time points/analyses) were sought or how results to collect were decided.
	10b	List and define all other variables for which data were sought (e.g. participant and intervention characteristics, funding sources). Describe any assumptions made about any missing or unclear information.	Reported – Methods, section 2.6 'Data Items,' p. 6 (study/participant characteristics, protocols, outcomes/time points, variances, adherence, withdrawals, adverse events, funding, conflicts of interest).

PRISMA 2020 Checklist

Section and Topic	Item #	Checklist item	Location where item is reported
Study risk of bias assessment	11	Specify the methods used to assess risk of bias in the included studies, including details of the tool(s) used, how many reviewers assessed each study and whether they worked independently, and if applicable, details of automation tools used in the process.	Reported – Methods, §2.7 'Risk of Bias Assessment,' p. 6 (Cochrane RoB 2, 5 domains, 2 independent reviewers, third-reviewer arbitration).
Effect measures	12	Specify for each outcome the effect measure(s) (e.g. risk ratio, mean difference) used in the synthesis or presentation of results.	Reported – Methods, §2.10 'Data Synthesis and Statistical Analysis,' p. 7 (mean difference and Hedges' g SMD; direction of effect defined).
Synthesis methods	13a	Describe the processes used to decide which studies were eligible for each synthesis (e.g. tabulating the study intervention characteristics and comparing against the planned groups for each synthesis (item #5)).	Partially reported – Methods, §2.10 'Data Synthesis and Statistical Analysis,' p. 7, specifies that pooling of crossover/limb-randomized data required a paired estimate and variance, but does not describe a formal tabulation of intervention characteristics against planned synthesis groups.
	13b	Describe any methods required to prepare the data for presentation or synthesis, such as handling of missing summary statistics, or data conversions.	Reported – Methods, §2.10 'Data Synthesis and Statistical Analysis,' p. 7 (no imputation of missing change-score SDs; Hedges' g used to combine differently scaled EMG data).
	13c	Describe any methods used to tabulate or visually display results of individual studies and syntheses.	Reported – Table 1 (pp. 9–10, study-level), Table 2 (p. 19, GRADE summary), and forest plots, Figures 3–5 (pp. 17–18).
	13d	Describe any methods used to synthesize results and provide a rationale for the choice(s). If meta-analysis was performed, describe the model(s), method(s) to identify the presence and extent of statistical heterogeneity, and software package(s) used.	Reported – Methods, §2.10 'Data Synthesis and Statistical Analysis,' p. 7 (REML random-effects model, Hartung–Knapp CIs; τ^2, Cochran's Q, I^2; software: Python 3.12.13, NumPy, SciPy, Matplotlib).
	13e	Describe any methods used to explore possible causes of heterogeneity among study results (e.g. subgroup analysis, meta-regression).	Not reported – no subgroup analysis or meta-regression was conducted or described (too few studies per outcome).
	13f	Describe any sensitivity analyses conducted to assess robustness of the synthesized results.	Not reported – no sensitivity analyses were conducted or described.
Reporting bias assessment	14	Describe any methods used to assess risk of bias due to missing results in a synthesis (arising from reporting biases).	Reported – Methods, §2.8 'Reporting Bias Assessment,' p. 7 (funnel plot/small-study tests not performed, <10 studies per outcome; considered qualitatively).
Certainty assessment	15	Describe any methods used to assess certainty (or confidence) in the body of evidence for an outcome.	Reported – Methods, §2.9 'Certainty of Evidence,' p. 7 (GRADE, 2 independent reviewers, Summary of Findings table).
**RESULTS**			
Study selection	16a	Describe the results of the search and selection process, from the number of records identified in the search to the number of studies included in the review, ideally using a flow diagram.	Reported – Results, section 3.1 'Study Selection,' p. 8 (search yield now broken down per database), and Figure 1, PRISMA flow diagram (p. 8).

PRISMA 2020 Checklist

Section and Topic	Item #	Checklist item	Location where item is reported
	16b	Cite studies that might appear to meet the inclusion criteria, but which were excluded, and explain why they were excluded.	Partially reported – Results, section 3.1 'Study Selection,' p. 8, gives counts of full-text exclusions by reason (e.g., 5 inappropriate outcomes, 2 study design, 7 unfinished/protocol-only, 1 non-English, 2 grey literature) but does not cite the individual excluded studies.
Study characteristics	17	Cite each included study and present its characteristics.	Reported – Results, section 3.2 'Study Characteristics,' p. 9, and Table 1 (pp. 9–10).
Risk of bias in studies	18	Present assessments of risk of bias for each included study.	Reported – Results, section 3.3 'Risk of Bias,' p. 11, and Figure 2 (p. 11).
Results of individual studies	19	For all outcomes, present, for each study: (a) summary statistics for each group (where appropriate) and (b) an effect estimate and its precision (e.g. confidence/credible interval), ideally using structured tables or plots.	Partially reported – section 3.4 'Narrative Synthesis' subsections (pp. 11–16) give study-level point estimates in text, generally without confidence intervals; Figures 3–5 (pp. 17–18) show individual study estimates with CIs, but only for the 3 quantitatively pooled outcomes (CSA, EMG RMS, torque-based strength).
Results of syntheses	20a	For each synthesis, briefly summarise the characteristics and risk of bias among contributing studies.	Partially reported – Results, section 3.3 'Risk of Bias,' p. 11, states all 5 trials were rated 'some concerns' overall.
	20b	Present results of all statistical syntheses conducted. If meta-analysis was done, present for each the summary estimate and its precision (e.g. confidence/credible interval) and measures of statistical heterogeneity. If comparing groups, describe the direction of the effect.	Reported – section 3.5 'Quantitative Synthesis (Meta-Analysis)' (§3.5.1–§3.5.3), pp. 16–18, and Table 2 (p. 19): pooled MD/SMD, 95% CI, heterogeneity (I^2, τ^2, Q) and direction of effect for CSA, EMG RMS, and torque-based strength; Figures 3–5.
	20c	Present results of all investigations of possible causes of heterogeneity among study results.	Not reported – no subgroup analysis or meta-regression results (see item 13e).
	20d	Present results of all sensitivity analyses conducted to assess the robustness of the synthesized results.	Not reported – no sensitivity analysis results (see item 13f).
Reporting biases	21	Present assessments of risk of bias due to missing results (arising from reporting biases) for each synthesis assessed.	Reported – Table 2 footnote, pp. 19–20 ('publication bias could not be assessed... too few studies contributed to each outcome').
Certainty of evidence	22	Present assessments of certainty (or confidence) in the body of evidence for each outcome assessed.	Reported – section 3.6 'Certainty of the Evidence (GRADE),' pp. 18–20, and Table 2 (p. 19): low/very low certainty for every outcome, with reasons for downgrading.
**DISCUSSION**			
Discussion	23a	Provide a general interpretation of the results in the context of other evidence.	Reported – Discussion, section 4.1 'Summary of Main Findings' (p. 21) and section 4.5 'Situating These Findings Within the Broader Literature' (pp. 22–23).

Section and Topic	Item #	Checklist item	Location where item is reported
	23b	Discuss any limitations of the evidence included in the review.	Reported – Discussion, section 4.7 'Strengths and Limitations,' p. 23, first paragraph (small evidence base, heterogeneous protocols).
	23c	Discuss any limitations of the review processes used.	Reported – Discussion, section 4.7 'Strengths and Limitations,' pp. 23–24, second paragraph (English-language restriction, excluded reports, Google Scholar limited to 100 results, no separate trial-registry search).
	23d	Discuss implications of the results for practice, policy, and future research.	Reported – Discussion, section 4.6 'Certainty of the Evidence and Clinical Implications' (p. 23) and 4.8 'Future Research Directions' (p. 24).
**OTHER INFORMATION**			
Registration and protocol	24a	Provide registration information for the review, including register name and registration number, or state that the review was not registered.	Reported – Methods, section 2.1 'Protocol and Registration,' p. 4, and Abstract, p. 1 (PROSPERO CRD420251076162).
	24b	Indicate where the review protocol can be accessed, or state that a protocol was not prepared.	Reported – registration number given (PROSPERO CRD420251076162), p. 4.
	24c	Describe and explain any amendments to information provided at registration or in the protocol.	Not reported – no amendments to the registered protocol are described.
Support	25	Describe sources of financial or non-financial support for the review, and the role of the funders or sponsors in the review.	Reported – 'Funding' statement, p. 25: this research received no external funding.
Competing interests	26	Declare any competing interests of review authors.	Reported – 'Conflicts of Interest' statement, p. 25: the authors declare no conflicts of interest.
Availability of data, code and other materials	27	Report which of the following are publicly available and where they can be found: template data collection forms; data extracted from included studies; data used for all analyses; analytic code; any other materials used in the review.	Reported – 'Data Availability Statement,' p. 25: no new data were created or analyzed, data sharing not applicable. Analysis software is named in §2.10 (p. 7); no separate analytic-code availability statement is given. 'Supplementary Materials,' p. 24, notes Table S1 (this PRISMA 2020 checklist) as supporting information.

From: Page MJ, McKenzie JE, Bossuyt PM, Boutron I, Hoffmann TC, Mulrow CD, et al. The PRISMA 2020 statement: an updated guideline for reporting systematic reviews. BMJ 2021;372:n71. doi: 10.1136/bmj.n71
